# Ophiuroidea (Echinodermata) from coral reefs in the Mexican Pacific

**DOI:** 10.3897/zookeys.406.6306

**Published:** 2014-05-07

**Authors:** Rebeca Granja–Fernández, María D. Herrero-Pérezrul, Ramón A. López-Pérez, Luis Hernández, Fabián A. Rodríguez-Zaragoza, Robert Wallace Jones, Rubén Pineda-López

**Affiliations:** 1 Facultad de Ciencias Naturales, Universidad Autónoma de Querétaro. Av. de las Ciencias s/n, Juriquilla, 76230, Querétaro, México; 2 Centro Interdisciplinario de Ciencias Marinas. Instituto Politécnico Nacional. Av. Instituto Politécnico Nacional s/n, col. Playa Palo de Santa Rita, 23096, La Paz, B.C.S., México; 3 Departamento de Hidrobiología, División CBS, UAM-Iztapalapa. Av. San Rafael Atlixco 186, Col. Vicentina, 09340, AP 55-535, DF, México; 4 Departamento Académico de Biología Marina, Universidad Autónoma de Baja California Sur. Carretera al sur km. 5.5, 23080, AP 19-B, La Paz, B.C.S, México; 5 Laboratorio de Ecosistemas Marinos y Acuicultura, Centro Universitario de Ciencias Biológicas y Agropecuarias, Universidad de Guadalajara. Carretera a Nogales km 15.5, Las Agujas-Nextipac, 45110, Zapopan, Jalisco, México

**Keywords:** Brittle stars, checklist, identification key, Mexico, reef zones

## Abstract

There are numerous and important coral reefs in the Mexican Pacific, but scarce studies of brittle stars conducted in these ecosystems. In this regard, this work provides the first annotated checklist of brittle stars associated with coral communities and reefs in the Mexican Pacific and an illustrated key to identify the species. We also provide taxonomic descriptions, spatial and bathymetric distributions and some important remarks of the species. We report a total of 14 species of brittle stars belonging to nine genera and seven families. *Ophiocnida hispida* in Jalisco, *Ophiophragmus papillatus* in Guerrero, and *Ophiothrix* (*Ophiothrix*) *spiculata* and *Ophiactis simplex* in Colima are new distribution records. The record of *O. papillatus* is remarkable because the species has not been reported since its description in 1940. The brittle stars collected in this study, represent 22.2% of the total species previously reported from the Mexican Pacific. Presently, anthropogenic activities on the coral reefs of the Mexican Pacific have increased, thus the biodiversity of brittle stars in these ecosystems may be threatened.

## Introduction

Coral reefs are among the most diverse and complex of all ecosystems and their diversity is comparable to that of the most diverse terrestrial habitats (Reaka-Kudla 1997). Structurally, coral reefs are composed of highly complex three dimensional environments which provide a variety of complex physical structures for the movement, concealment, escape, refuge, reproduction, breeding and feeding of numerous marine animals (Spalding et al. 2001). Coral reef biodiversity is commonly dominated by invertebrates, with the Mollusca, Arthropoda and Echinodermata generally being the most diverse and numerous phyla (Stella et al. 2011).

Echinoderms are conspicuous invertebrates on coral reefs and greatly contribute to the overall ecosystem biomass. Some species play pivotal keystone roles, and changes in their abundance can have large-scale effects on reef community structure and ecosystem functioning (Birkeland 1989, Glynn and Enochs 2011). Amongst the echinoderms, brittle stars are well represented in coral reef ecosystems and most species are associated with various invertebrate hosts or microhabitats (Glynn and Enochs 2011). Some studies reveal that coral reef-associated brittle stars can occur in densities from 20 to 15,000 individuals/m^2^ (Chartock 1983, Hendler et al. 1995). Brittle stars feature in the diets of many coral reef fishes. Randall (1967) estimated that brittle stars occurred in the stomachs of 33 Caribbean reef fish, constituting, in some cases, up to 16% of stomach contents. Birkeland (1989) suggests that in virtue of the abundance, biomass and energy flow, it is assumed that brittle stars are influential in coral-reef trophic functioning. For example, Pomory and Lawrence (2001) suggest brittle stars regeneration can contribute from 0.05 to 0.07% of the net primary production on a reef, providing an important biomass flow to higher trophic levels.

Coral reefs of the Pacific coast of Mexico are considered among the most important in the eastern Pacific (Glynn and Ault 2000). Of these, the coral reefs located in the states of Nayarit, Guerrero and Oaxaca are considered the most important and well developed. Reefs of the coast of the states of Jalisco, Colima and Michoacán are numerous eventhough they are small communities and occur in patches (Reyes-Bonilla 2003, López-Pérez et al. 2012, Reyes-Bonilla et al. 2013). Mexican Pacific coral reefs are mainly constructed by species of the genera *Pocillopora*, *Porites* and *Pavona* (Reyes-Bonilla 2003). From the Pacific coast of Mexico, there are a large number of studies regarding echinoderms (e.g. Honey-Escandón et al. 2008, Solís-Marín et al. 2013 and references there in); nevertheless, studies of brittle stars in, or associated with coral reefs in this area are limited (Cintra-Buenrostro et al. 1998, Benítez-Villalobos 2001, Zamorano and Leyte-Morales 2005, Ríos-Jara et al. 2008, 2013, Granja-Fernández and López-Pérez 2011), and most are confined to specific reef localities (e.g. La Entrega, Oaxaca).

Recently it has been suggested that the publication of taxonomic studies and guides of species should be a priority for research and environmental management since they enrich the knowledge of regional biodiversity (Costello et al. 2010), particularly in poorly prospected and potentially megadiverse areas such as the Mexican Pacific. The Tropical Mexican Pacific reefs are among the least prospected, but at the same time are considered among the most important in the Eastern Pacific (Glynn and Ault 2000). Although, the Ophiuroidea inhabiting the coral reefs of the Mexican Pacific have been the subject of research, most of these studies have had an ecological focus, and basic taxonomic baseline data and guides for identification of species are lacking. The aim of the present work is to provide an annotated checklist of brittle stars that inhabit these ecosystems and a key to identify species associated with coral reefs and coral communities.

## Methods

This project was part of a larger multidisciplinary and inter-institutional programme on the regarding fauna associated with coral reef zones along the Mexican Pacific. We sampled a total of 59 coral reefs of Mexico located along the coasts of the states of Nayarit (3), Jalisco (6), Colima (4), Michoacán (5), Guerrero (12) and Oaxaca (29), between 2007 and 2012 (Table 1).

**Table 1.** Geographic coordinates of prospected coral reefs from the Mexican Pacific. *Nay* Nayarit; *Jal* Jalisco; *Col* Colima; *Mich* Michoacán; *Gro* Guerrero; *Oax* Oaxaca.

Coral reef	State	Geographic coordinates
Las Monas	Nay	21°51'06"N, 105°52'47"W
Bahía Rabijuncos	Nay	21°50'35"N, 105°53'23"W
Las Pozas	Nay	21°50'29"N, 105°53'03"W
Pelícanos	Jal	19°33'27"N, 105°06'28"W
La Pajarera	Jal	19°33'24"N, 105°06'28"W
La Palma	Jal	19°33'02"N, 105°06'23"W
Isla Cocinas	Jal	19°32'46"N, 105°06'28"W
Cuastecomatito	Jal	19°13'58"N, 104°45'15"W
La Virgencita	Jal	19°14'09"N, 104°44'57"W
Carrizales	Col	19°05'48"N, 104°26'09"W
L’Recif	Col	19°06'01"N, 104°24'24"W
La Boquita	Col	19°06'08"N, 104°23'38"W
Punto B	Col	19°05'55"N, 104°23'24"W
Isla Pájaros	Mich	18°21'09"N, 103°31'09"W
Morro de Enmedio	Mich	18°21'11"N, 103°31'02"W
Faro de Bucerías	Mich	18°21'08"N, 103°30'58"W
Morro Chino	Mich	18°21'56"N, 103°08'10"W
Caleta de Campos	Mich	18°04'11"N, 102°44'37"W
Morro del Cerro Colorado	Gro	17°40'50"N, 101°39'32"W
Coral	Gro	17°40'31"N, 101°39'20"W
Playa Carey	Gro	17°40'31"N, 101°39'03"W
Punta Ixtapa	Gro	17°39'52"N, 101°38'11"W
Zacatoso	Gro	17°39'15"N, 101°37'19"W
El Chato	Gro	17°39'01"N, 101°37'17"W
Caleta de Chón	Gro	17°36'55"N, 101°33'17"W
El Yunque	Gro	17°36'59"N, 101°31'56"W
Manzanillo	Gro	17°37'10"N, 101°31'27"W
Morros de Potosí	Gro	17°31'59"N, 101°29'28"W
Palmitas	Gro	16°49'23"N, 99°54'44"W
El Ripial	Gro	16°49'19"N, 99°54'04"W
Playa Coral	Oax	15°51'40"N, 97°05'11"W
Carrizalillo	Oax	15°51'27"N, 97°04'47"W
Puerto Angelito	Oax	15°51'24"N, 97°04'26"W
El Zapatito	Oax	15°51'12"N, 97°04'19"W
Punto de Presión	Oax	15°51'10"N, 97°04'01"W
El Faro	Oax	15°51'24"N, 97°03'58"W
Mazunte	Oax	15°39'35"N, 96°33'17"W
Puerto Ángel	Oax	15°39'49"N, 96°29'40"W
Estacahuite	Oax	15°40'06"N, 96°28'52"W
La Mina	Oax	15°40'24"N, 96°28'38"W
Boquilla	Oax	15°40'48"N, 96°27'55"W
Tijera	Oax	15°41'15"N, 96°26'31"W
Salchi	Oax	15°40'58"N, 96°21'49"W
San Agustín	Oax	15°41'14"N, 96°14'13"W
Riscalillo	Oax	15°41'48"N, 96°13'29"W
Jicaral	Oax	15°42'01"N, 96°12'51"W
Dos Hermanas	Oax	15°42'04"N, 96°12'35"W
Harrys	Oax	15°42'10"N, 96°12'20"W
Pomelo	Oax	15°42'27"N, 96°11'33"W
Copal	Oax	15°42'57"N, 96°10'38"W
Isla Cacaluta	Oax	15°43'12"N, 96°09'47"W
Maguey	Oax	15°43'48"N, 96°08'54"W
Órgano	Oax	15°44'11"N, 96°08'37"W
Violín	Oax	15°44'15"N, 96°08'01"W
La Entrega	Oax	15°44'39"N, 96°07'43"W
Manzanilla	Oax	15°46'00"N, 96°05'57"W
Isla Montosa	Oax	15°45'55"N, 96°05'01"W
Guerrilla	Oax	15°46'12"N, 96°04'33"W
Copalita	Oax	15°46'51"N, 96°03'35"W

Collections were carried out in a variety of substrata in or adjacent to coral reefs (live and dead stony corals, gorgonians, rock, sand, algae, rhodoliths, and sponges) and at different depths (1-26 m). The sampling was carried out by hand collection while SCUBA diving. Once in the laboratory, the collected ophiuroids were anesthetized using magnesium chloride buffered seawater in order to prevent autotomy. Finally, the specimens were fixed and preserved in 70% ethanol.

The specimens were identified using the works of Müller and Troschel (1842), Le Conte (1851), Lütken (1856, 1859), Lyman (1860, 1874), Verrill (1867), Ziesenhenne (1940), Fell (1960) and Hendler et al. (1995). The valid names agree with Stöhr and O’Hara (2013) and were arranged systematically according to Smith et al. (1995). The specimens were deposited in the Colección Nacional de Equinodermos “Dra. María Elena Caso Muñoz”, at UNAM (ICML-UNAM), Distrito Federal, Mexico, and in the Colección de Equinodermos de la Universidad del Mar (MHN), Puerto Ángel, México.

## Results

We identified a total of 14 species of Ophiuroidea (approximately 4,000 individuals) from the coral reefs from the Mexican Pacific, distributed in seven families (Amphiuridae Ljungman, 1867; Ophiotrichidae Ljungman, 1867; Ophiactidae Matsumoto, 1915; Ophionereididae Ljungman, 1867; Ophiocomidae Ljungman, 1867; Ophiodermatidae Ljungman, 1867; Ophiolepididae Ljungman, 1867) and nine genera (*Ophiocnida* Lyman, 1865; *Ophiophragmus* Lyman, 1865; *Ophiothrix* Müller & Troschel, 1840; *Ophiothela* Verrill, 1867; *Ophiactis* Lütken, 1856; *Ophionereis* Lütken, 1859; *Ophiocoma* L. Agassiz, 1835; *Ophioderma* Müller & Troschel, 1840; *Ophiolepis* Müller & Troschel, 1840). The families with the highest number of species were Ophiotrichidae (3) and Ophiodermatidae (3).

### Key to the identification of the families, genera and species of brittle stars (Ophiuroidea) from coral reefs of the Mexican Pacific

**Table:** 

1a	Cluster of dental papillae at the apex of the jaw (Fig. 2F)	2
1b	Dental papillae absent (Fig. 1F)	6
2a	Disk covered with granules (Fig. 5J). Oral papillae present on the sides of the jaw (Fig. 5F). Arm spines stout and erect (Figs 5B, C)	family Ophiocomidae, genus *Ophiocoma*, 3
2b	Disk covered with spines, stumps (Fig. 2D) and/or grains (Fig. 3D). Oral papillae absent (Fig. 2F). Arm spines long and erect with a glassy, translucent appearance (Fig. 2H, I)	family Ophiotrichidae, 4
3a	Oral shield large and oblong. Three and four arm spines, alternating. Two tentacle scales in all the joints. Dark brown-black in color (Figs 5A-F)	*Ophiocoma aethiops*
3b	Oral shields rounded. Five-seven arm spines. Two tentacle scales on first two or three joints, the remaining with only one. Brown in color. Ventral side of arms with a longitudinal stripe (Figs 5G–L)	*Ophiocoma alexandri*
4a	Disk with spines and stumps. Tentacle scales present (Fig. 2F, I)	genus *Ophiothrix*, 5
4b	Disk covered by scattered grains of different sizes. Tentacle scales absent. Six arm spines with well-developed hooks at the tip. Six rolled up arms. Rosaceous in color (Fig. 3A–F)	genus *Ophiothela*, *Ophiothela mirabilis*
5a	Disk covered only with spinules. Five-seven arm spines not serrated. Dorsal side of the disk dark indigo-bluish (Fig. 2A–F)	*Ophiothrix (Ophiothrix) rudis*
5b	Disk covered with spinules and/or spines. Seven-eight arm spines serrated and hyaline. Dorsal side may present different colors (blue, purple, yellow, orange, brown) (Fig. 2G–L)	*Ophiothrix (Ophiothrix) spiculata*
6a	Paired block-like infradental papillae at the apex of the jaw (Fig. 1F). Oral papillae present on sides of the jaw. Short and erect arm spines (Fig. 1B, C)	family Amphiuridae, 7
6b	Infradental papillae not block-like. Oral papillae and teeth present (Fig. 6F)	8
7a	Dorsal side of disk with numerous scattered spines and imbricated scales. Madreporite plate evident. Disk brown. Arms light brown with irregularly distributed transversal lines (Fig. 1A–F)	genus *Ophiocnida*, *Ophiocnida hispida*
7b	Dorsal side of disk with imbricated scales and few papillae. Primary plates present. Papillae around the margin of the disk. Disk white-creamy. Arms brown with black spots, and a creamy middle longitudinal line (Fig. 1G–L)	genus *Ophiophragmus*, *Ophiophragmus papillatus*
8a	Disk covered with granules (Fig. 6D). Arms stout (Fig. 6G). Arm spines numerous, short and appressed to the side of the arm (Figs 6B, C). Four bursal slits per interradius (Fig. 6E)	family Ophiodermatidae, genus *Ophioderma*, 9
8b	Disk covered with scales and/or spines (Fig. 3J). Arms spines erect (Fig. 3H, I). Two bursal slits per interradius (Fig. 7K)	11
9a	Dorsal arm plates generally subdivided in smaller pieces (Fig. 6H)	10
9b	Radial shields covered. Oral shields heart-shaped. Adoral shields uncovered. Dorsal arm plates not subdivided in smaller pieces. Ten to eleven arm spines. Color brown. Dorsal arm plates with transversal white bands (Fig. 7A-F)	*Ophioderma* sp. 1
10a	Radial shields uncovered. Oral shields oval. Adoral shields covered. Few dorsal arm plates divided in two pieces can be present. Ten to eleven arm spines. Dorsal side brownish in color. Arms with dark and light bands (Fig. 6A–F)	*Ophioderma panamensis*
10b	Radial shields uncovered. Oral shields heart-shaped. Adoral shields covered. Some dorsal arm plates divided in more than two pieces. Six to seven arm spines. Dorsal side brown chocolate in color with irregular black rings on the disk (Fig. 6G–L)	*Ophioderma teres*
11a	Disk covered with scales often carrying scattered spines. Oral papillae not forming a continuous series along the margin of the jaw (Fig. 4F). Arm spines short and erect. Accessory dorsal arm plates absent (Fig. 3H). Organisms with 6 or fewer arms (Fig. 3G)	family Ophiactidae, genus *Ophiactis*, 12
11b	Disk covered only with scales (Fig. 4J). Accessory dorsal arm plates present (Figs 4H, 7H). Oral papillae forming a continuous series along the margin of the jaw (Fig. 4L)	13
12a	Disk and margin of the disk with spines. Radial shields large. Two small oral papillae on each side of jaw. Rugose-spinulose arm spines. Five or six arms. Greenish-brown in color (Figs 3G-L)	*Ophiactis savignyi*
12b	Disk with scattered spines. Radial shields small. One large oral papillae on each side of the jaw. Smooth arm spines. Five arms. Brown in color (Fig. 4A–F)	*Ophiactis simplex*
13a	Disk with small and imbricating scales. Radial shields small. Accessory dorsal arm plates large. Arm spines short and erect. Four oral papillae on each side of the jaw. Disk brown with darker spots. Arms with dark dorsal plates every fourth or fifth plate (Fig. 4G–L)	family Ophionereididae, genus *Ophionereis*, *Ophionereis annulata*
13b	Disk with groups of small scales surrounding larger scales. Radial shields small with a furrow parallel to the margin. Accessory dorsal arm plates small. Arm spines appressed to the side of the arm. Light brown in color. Dorsal arm plates with dark bands (Fig. 7G–L)	family Ophiolepididae, genus *Ophiolepis*, *Ophiolepis pacifica*

Annotated list of the species of brittle stars collected in coral reefs from the Mexican Pacific. In the collected material section, data within parentheses are: 1) number of specimens, 2) substrate, 3) depth, and 4) collection date. In the description of the species, the abbreviation “dd” refers to disk diameter (mm).

### Phyllum Echinodermata Brugiére, 1791
Class Ophiuroidea Gray, 1840
Order Ophiurida Müller & Troschel, 1840

#### Family Amphiuridae Ljungman, 1867

##### *Ophiocnida* Lyman, 1865

###### Ophiocnida hispida

(Le Conte, 1851)

http://species-id.net/wiki/Ophiocnida_hispida

Figure 1A–F

####### Description.

**Figure 1.**
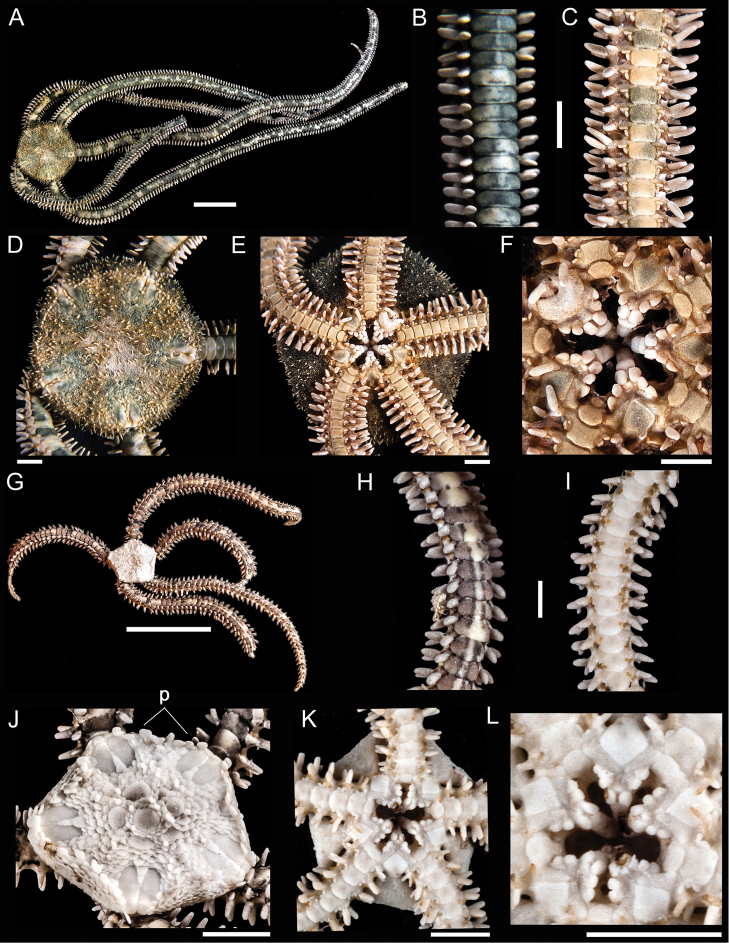
*Ophiocnida hispida*. **A** dorsal view. Scale bar = 5 mm **B** dorsal view of the arm **C** ventral view of the arm **D** dorsal view of the disk **E** ventral view of the disk **F** jaw. Scale bar = 1 mm. *Ophiophragmus papillatus* **G** dorsal view. Scale bar = 5 mm **H** dorsal view of the arm **I** ventral view of the arm **J** dorsal view of the disk (p = papillae around the margin of the disk) **K** ventral view of the disk **L** jaw. Scale bar = 1 mm.

Disk rounded (dd = 6 to 7.6 mm) and covered with imbricated scales bearing pointed scattered spines. Radial shields narrow and separated by a row of scales which are larger than those of the disk (Fig. 1D).Ventral interradius with smaller imbricated scales and bearing scattered spines (Fig. 1E). Oral shields diamond shaped, with rounded angles. Adoral shields triangular and not meeting within. Three papillae on each side of the jaw; two outer ones rounded and the innermost one being the largest. The madreporite is evident (Fig. 1F). Dorsal arm plates wider than long, with the corners rounded (Fig. 1B). Ventral arm plates rectangular, wider than long; outer and inner sides slightly curved. Three blunt, cylindrical and short arm spines of nearly equal length. Two small tentacle scales forming a right angle to each other (Fig. 1C). Color of the disk brown (Fig. 1A); the color in the ventral side of the disk is straw-brown (Fig. 1E). Arms straw colored with irregular transversal lines (Fig. 1B). Madreporite with a lighter color (Fig. 1F).

####### Distribution.

From USA (California) to Panama (McClendon 1909, Alvarado et al. 2010). In Mexico, from the Gulf of California (Baja California Sur, Sonora), Guerrero and Oaxaca (Solís-Marín et al. 2005, Honey-Escandón et al. 2008, Granja-Fernández and López-Pérez 2012). Depth 0-794 m (Maluf 1988). In this study, *Ophiocnida hispida* was collected in coral reefs from Jalisco and Oaxaca at 9.1 m depth.

####### Remarks.

There are five species of the genus *Ophiocnida* world-wide (including *Ophiocnida hispida*): *Ophiocnida loveni* (Ljungman, 1867), *Ophiocnida scabra* Lyman, 1879, *Ophiocnida scabriuscula* (Lütken, 1859) and *Ophiocnida californica* Ziesenhenne, 1940 (Stöhr and O’Hara 2013). *Ophiocnida hispida* and *Ophiocnida californica* are the only species reported from the Eastern Pacific; but *Ophiocnida californica* is confined to the Gulf of California. Ziesenhenne (1940) reports that both species can be distinguished by the size of the oral shields, with radial shields in contact for half of their length, the divided dorsal arm plates in *Ophiocnida californica*, and the arm length which is five times the disk diameter in *Ophiocnida californica* and eight to ten in *Ophiocnida hispida*. In the present study, *Ophiocnida hispida* was found buried exclusively in sand while other species of the genus, *Ophiocnida scabriuscula* and *Ophiocnida loveni* were reported from seagrass habitats, calcareous algae or under rocks (Hendler et al. 1995, de Barros-Lima and Banja-Fernandes 2009). *Ophiocnida hispida* is hard to manipulate because it can autotomize both the disk and the arms very easily. Koehler (1907) indicates that *Ophiocnida scabriuscula* can regenerate their disk and possess typical spines and radial shields of the genus. Future studies must be carried out to elucidate if this capability and morphology are shared with other members of the genus *Ophiocnida*, such as *Ophiocnida hispida*. The record of *Ophiocnida hispida* is new for Jalisco.

####### Collected material.

**JALISCO:** **Cuastecomatito** (1 specimen, sand, 30/09/2010, ICML-UNAM 10334).

**OAXACA:** **La Mina** (4 specimens, sand, 17/04/2008, MHN 005-4351); **Órgano** (2 specimens, sand, 08/08/2011, ICML-UNAM 10428); **Copalita** (1 specimen, sand, 9.1 m, 18/05/2012, ICML-UNAM 10526).

##### *Ophiophragmus* Lyman, 1865

###### Ophiophragmus papillatus

Ziesenhenne, 1940

http://species-id.net/wiki/Ophiophragmus_papillatus

Figure 1G–L

####### Description.

Disk rounded (dd = 1.7 to 2.7 mm). Disk covered with small and imbricated scales. There is a central primary plate surrounded by five separated smaller scales. There are a few blunt papillae in the center of the disk. The disk margin is composed of small and rounded papillae. Radial shields separated proximally by two large scales and distally in contact (Fig. 1J).Ventral side of the disk covered by imbricated and finer scales than those on the dorsal side (Fig. 1K). Oral shields diamond-shaped. Adoral shields meeting within. Three rounded oral papillae on each side of the jaw, apical papillae the largest and thickest. Four teeth (Fig. 1L). Dorsal arm plates rounded, wider than long (Fig. 1H). Ventral arm plates quadrangular with rounded margins, wider than long. Three blunt, thick and small arm spines; upper spine is the most robust. Two small tentacle scales perpendicular to each other (Fig. 1I). Dorsal side of the disk creamy-white. Dorsal arm plates brown with black spots, and with a creamy middle longitudinal line along the arms (Fig. 1G, H). Ventral side uniformly creamy-white (Fig. 1K).

####### Distribution.

Tangola Island, Tangola-Tangola Bay, Oaxaca, Mexico (Station 260–34) (Ziesenhenne 1940). In this study, *Ophiophragmus papillatus* was collected on coral reefs from Guerrero and Oaxaca at a depth of 6.4 m.

####### Remarks.

The collection of *Ophiophragmus papillatus* in La Mina, Oaxaca and Palmitas, Guerrero is remarkable because the species had not been reported since its description by Ziesenhenne in 1940. This study reports a range extension of almost 410 km north of the type locality (Tangolunda, Mexico). We collected three organisms which perfectly match the original description provided by Ziesenhenne (1940). These specimens have a similar disk diameter to the holotype, which is 3.6 mm (Ziesenhenne 1940); the specimen from Palmitas has a disk diameter of 2.7 mm, whereas the specimens from La Mina have disk diameters of 1.7 mm and 2.4 mm. The two smaller specimens presented the color pattern mentioned in the description but the brown color of the dorsal arm plates was less evident than in the largest specimen. *Ophiophragmus papillatus* was collected in rock and coral. Other members of the genus *Ophiophragmus* inhabit sand, soft mud, rock, seagrass, mangroves and reefs (Nielsen 1932, Hendler et al. 1995). *Ophiophragmus papillatus* is a new record distribution for the state of Guerrero.

####### Collected material.

**GUERRERO:** **Palmitas** (1 specimen, rock, 6.4 m, 20/11/2011, ICML-UNAM 10448).

**OAXACA:** **La Mina** (2 specimens, coral, 19/02/2009, ICML-UNAM 10583).

#### Family Ophiotrichidae Ljungman, 1867

##### *Ophiothrix* Müller & Troschel, 1840

###### Ophiothrix (Ophiothrix) rudis

Lyman, 1874

http://species-id.net/wiki/Ophiothrix_rudis

Figure 2A–F

####### Description.

**Figure 2.**
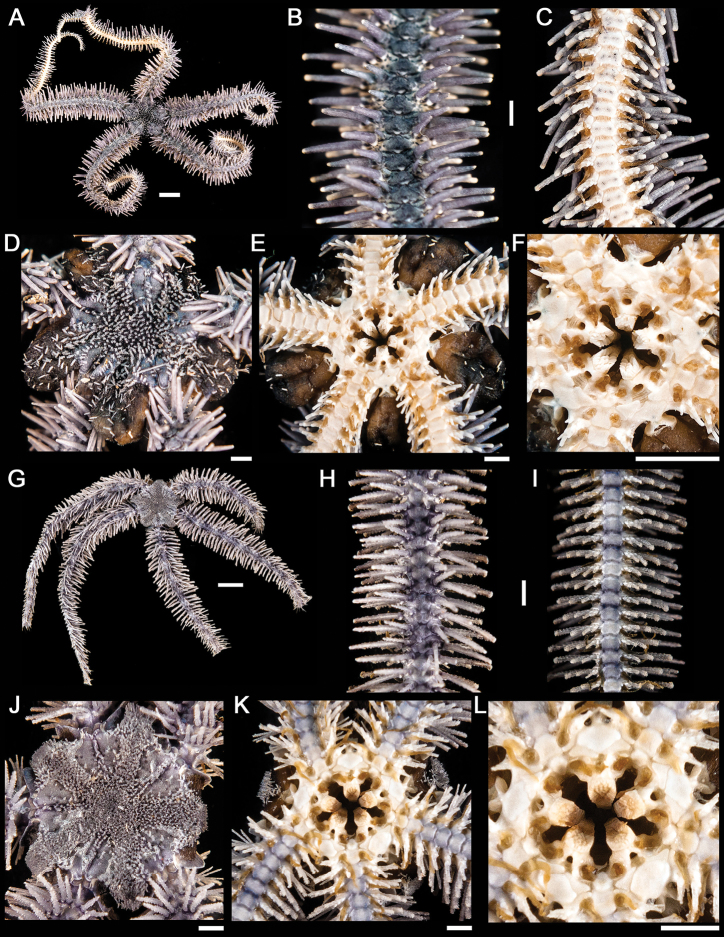
*Ophiothrix (Ophiothrix) rudis*. **A** dorsal view. Scale bar = 5 mm **B** dorsal view of the arm **C** ventral view of the arm **D** dorsal view of the disk **E** ventral view of the disk **F** jaw. Scale bar = 1 mm. *Ophiothrix (Ophiothrix) spiculata* **G** dorsal view. Scale bar = 5 mm **H** dorsal view of the arm **I** ventral view of the arm **J** dorsal view of the disk **K** ventral view of the disk **L** jaw. Scale bar = 1 mm.

Disk rounded and bearing spinules (dd = 2.4 to 11 mm). Radial shields completely naked and separated by a line of multifid spinules (Fig. 2D).Ventral side of the disk mostly naked with scattered multifid spinules (Fig. 2E). Oral shields rounded diamond-shape, wider than long. Adoral shields narrowed and slightly separated. Oral papillae lacking. About 35 dental papillae per jaw (Fig. 2F). Dorsal arm plates wider than long, triangular or fan shaped with rounded corners; a spot distally on each arm plate (Fig. 2B). Ventral arm plates quadrangular with rounded corners. Five to seven stout, blunt, rounded, and not serrated arm spines; the two upper ones being the longest. A single, rounded, and very small tentacle scale (Fig. 2C). The color of the dorsal side of the disk is dark indigo-bluish and the ventral side is pale yellow-cream. Spines are bluish (Fig. 2A). A pale spot may be present distal to the radial shields.

####### Distribution.

From California, USA to Oaxaca, Mexico (Lyman 1874, McClendon 1909, Nielsen 1932, Luke 1982). In Mexico, this species is reported from the Gulf of California, and from the coasts of the states of Baja California Sur, Nayarit, Michoacán, Guerrero and Oaxaca (Solís-Marín et al. 2005, Honey-Escandón et al. 2008, Granja-Fernández and López-Pérez 2011). Depth 0-64 m (Maluf 1988). In this study, *Ophiothrix (Ophiothrix) rudis* was collected on coral reefs from Michoacán, Guerrero and Oaxaca, between depths of 1.5 and 13.5 m.

####### Remarks.

*Ophiothrix (Ophiothrix) rudis* was found living on stony coral, dead coral, rocks, algae and on sponges. Boolotian and Leighton (1966) collected *Ophiothrix (Ophiothrix) rudis* from Palos Verdes, California in rocks and coarse sand. Although we made considerable effort to collect brittle stars from sand, we never found this species associated with this substrate. This species is easily distinguished from *Ophiothrix (Ophiothrix) spiculata* by using the following characteristics of *Ophiothrix (Ophiothrix) rudis*: arms not serrated, the disk only bears spinules which are larger than on *Ophiothrix (Ophiothrix) spiculata*, and the color pattern of the disk and on the arms. *Ophiothrix (Ophiothrix) rudis* is not as conspicuous or numerous as *Ophiothrix (Ophiothrix) spiculata* in the study area. Benítez-Villalobos (2001) observed that *Ophiothrix (Ophiothrix) rudis* is one of the less abundant species in La Entrega reef in Oaxaca, Mexico; we did not find the species in this coral reef area.

####### Collected material.

**MICHOACÁN:** **Caleta de Campos** (7 specimens, stony coral, 2.5 m, 22/02/2010, ICML-UNAM 10245).

**GUERRERO:** **Morro del Cerro Colorado** (14 specimens, stony coral, 1.5 m, 30/11/2010, MHN 005-4471; 5 specimens, stony coral, 3 m, 30/11/2010, MHN 005-4454; 1 specimen, rock, 30/11/2010, ICML-UNAM 10359; 2 specimens, rock, 4.5 m, 23/11/2011, ICML-UNAM 10477; 5 specimens, dead coral, 3 m, 31/05/2012, ICML-UNAM 10556; 3 specimens, rock, 5.5 m, 31/05/2012, ICML-UNAM 10557); **Carey** (1 specimen, rock, 4 m, 23/11/2011, ICML-UNAM 10495); **Punta Ixtapa** (4 specimens, stony coral, 4.2 m, 19/02/2010, ICML-UNAM 10255); **Zacatoso** (6 specimens, stony coral, 9.1 m, 01/12/2010, MHN 005-4431; 1 specimen, algae, 7.5 m, 01/12/2010, ICML-UNAM 10375; 2 specimens, sponge, 01/12/2010, ICML-UNAM 10371; 1 specimen, rock, 9.1 m, 01/06/2012, ICML-UNAM 10572; 1 specimen, sponge, 9.1 m, 01/06/2012, ICML-UNAM 10573); **Caleta de Chón** (6 specimens, stony coral, 4.6 m, 02/12/2010, MHN 005-4451; 2 specimens, stony coral, 6.1 m, 02/12/2010, MHN 005-4439; 2 specimens, stony coral, 7.6 m, 02/12/2010, MHN 005-4468; 1 specimen, rock, 02/12/2010, ICML-UNAM 10388; 1 specimen, algae, 4.5 m, 02/12/2010, ICML-UNAM 10385; 3 specimens, rock, 5 m, 22/11/2011, ICML-UNAM 10472); **El Yunque** (1 specimen, rock, 04/12/2010, ICML-UNAM 10403; 3 specimens, sponge, 5.5 m, 04/12/2010, ICML-UNAM 10404; 1 specimen, rock, 8 m, 24/11/2011, ICML-UNAM 10500); **Manzanillo** (1 specimen, rock, 05/11/2009, ICML-UNAM 10237; 1 specimen, stony coral, 3 m, 04/12/2010, MHN 005-4460; 1 specimen, dead coral, 6.1 m, 30/05/2012, ICML-UNAM 10542; 3 specimens, rock, 6.1 m, 30/05/2012, ICML-UNAM 10543; 2 specimens, sponge, 6.1 m, 30/05/2012, ICML-UNAM 10544); **Coral** (5 specimens, rock, 5 m, 23/11/2011, ICML-UNAM 10489).

**OAXACA:** **Puerto Angelito** (1 specimen, rock, 10 m, 23/04/2012, ICML-UNAM 10506); **El Zapatito** (3 specimens, sponge, 23/04/2009, ICML-UNAM 10220); **El Faro** (1 specimen, rock, 13.5 m, 24/04/2012, ICML-UNAM 10512); **Salchi** (1 specimens, stony coral, 6.1 m, 26/03/2010, MHN 005-4413); **Riscalillo** (1 specimen, rock, 04/12/2008, MHN 005-4348); **Copal** (8 specimens, sponge, 9.1 m, 21/10/2011, ICML-UNAM 10440); **Manzanilla** (1 specimen, rock, 03/12/2008, MHN 005-4340; 2 specimens, dead coral, 3 m, 18/05/2012, ICML-UNAM 10522); **Isla Montosa** (2 specimens, stony coral, 5.8 m, 22/02/2010, MHN 005-4385; 5 specimens, stony coral, 6.1 m, 22/02/2010, MHN 005-4381); **Guerrilla** (24 specimens, rock, 4.9 m, 18/05/2012, ICML-UNAM 10534); **Copalita** (1 specimen, rock, 9.1 m, 18/05/2012, ICML-UNAM 10527; 2 specimens, sponge, 9.1 m, 18/05/2012, ICML-UNAM 10528).

###### Ophiothrix (Ophiothrix) spiculata

Le Conte, 1851

http://species-id.net/wiki/Ophiothrix_spiculata

Figure 2G–L

####### Description.

Disk circular with interradial protruding (dd = 1.2 to 10.2 mm). Disk totally covered with short multifid spinules and spines. Radial shields nearly touching distally and separated by a row of scales and spinules (Fig. 2J).Ventral side of the disk sparsely covered by small spines and scales (Fig. 2K). Oral shields wider than long and diamond shaped with lateral sides lobate; madreporite evident. Adoral shields triangular, small and in contact. Oral papillae lacking. A dental papillae cluster at the ventral apex of the jaw (Fig. 2L). Dorsal arm plates pentagonal with rounded edges (Fig. 2H). Ventral arm plates wider than long, wider on the distal edge. Up to eight serrated and hyaline arm spines; top-most spine is usually short, second or third are the longest. One small tentacle scale (Fig. 2I). Color of the disk bluish-purple (Fig. 2G). Mouth area whitish-yellow (Fig. 2K, L).

####### Distribution.

Bering Sea, USA (California), Mexico, El Salvador, Nicaragua, Costa Rica, Panama, Colombia, Peru, Chile, Galapagos Islands (Clark HL 1911, 1915, Hooker et al. 2005, Neira and Cantera 2005, Alvarado et al. 2010). In Mexico, from the Gulf of California (Baja California Sur, Sonora, Sinaloa), on the Pacific side of Baja California and Baja California Sur, Nayarit, Islas Marías, Jalisco, Michoacán, Guerrero and Oaxaca (Solís-Marín et al. 2005, Honey-Escandón et al. 2008, Granja-Fernández and López-Pérez 2011). Depth intertidal to 2059 m (Maluf 1988). In this study, *Ophiothrix (Ophiothrix) spiculata* was collected on coral reefs from Nayarit, Jalisco, Colima, Michoacán, Guerrero and Oaxaca, between 1.5 and 26 m depth.

####### Remarks.

*Ophiothrix (Ophiothrix) spiculata* is one of the most conspicuous and abundant species on the coral reefs of the Mexican Pacific. Numerous authors have reported that this species is very abundant in other areas such as California and Panama (Verrill 1867, McClendon 1909). Austin and Haldfield (1980) reported that densities of *Ophiothrix (Ophiothrix) spiculata* reach up to 80 individuals/0.1 m^2^ in a siltstone reef off Newport Bay, California. We observed that *Ophiothrix (Ophiothrix) spiculata* form massive aggregations; the advantages of which has been reported to be protection from predators, enhanced feeding ability in strong currents and to maximize fertilization during broadcast spawning (Hendler 1991). We found this species in live stony coral, dead coral, rocks, algae, rhodolith and on sponges. According to Maluf (1988), the species can inhabit coral, rocks, sponges, mangroves and sand. As in the same case of *Ophiothrix (Ophiothrix) rudis*, we did not find *Ophiothrix (Ophiothrix) spiculata* in sand. Besides the most common color pattern described above, we found some specimens completely orange and yellow collected on sponges and, brown in color which was mostly collected on rocks and dead coral. *Ophiothrix (Ophiothrix) spiculata* is a new record distribution for the state of Colima.

####### Collected material.

**NAYARIT:** **Las Monas** (4 specimens, stony coral, 17/06/2010, ICML-UNAM 10356; 1 specimen, rock, 09/05/2011, ICML-UNAM 10418); **Bahía Rabijuncos** (5 specimens, stony coral, 08/06/2011, ICML-UNAM 10432).

**JALISCO:** **Cuastecomatito** (1 specimen, rock, 30/09/2010, ICML-UNAM 10335); **Pelícanos** (3 specimens, stony coral, 29/09/2010, ICML-UNAM 10316; 2 specimens, rock, 29/09/2010, ICML-UNAM 10317); **La Pajarera** (1 specimen, stony coral, 29/09/2010, ICML-UNAM 10305; 1 specimen, rock, 29/09/2010, ICML-UNAM 10306); **La Palma** (2 specimens, stony coral, 29/09/2010, ICML-UNAM 10322; 1 specimen, rock, 29/09/2010, ICML-UNAM 10323); **La Virgencita** (1 specimen, stony coral, 30/09/2010, ICML-UNAM 10339).

**COLIMA:** **La Boquita** (1 specimen, stony coral, 2.5 m, 26/02/2010, ICML-UNAM 10252; 5 specimens, stony coral, 01/10/2010, ICML-UNAM 10354); **Carrizales** (1 specimen, 01/10/2010, ICML-UNAM 10348).

**MICHOACÁN:** **Faro de Bucerías** (4 specimens, 28/09/2010, ICML-UNAM 10295); **Isla Pájaros** (3 specimens, stony coral, 28/09/2010, ICML-UNAM 10288; 3 specimens, dead coral, 28/09/2010, ICML-UNAM 10283); **Morro Chino** (5 specimens, stony coral, 4.3 m, 23/02/2010, ICML-UNAM 10246); **Morro de Enmedio** (1 specimen, stony coral, 3.7 m, 24/02/2010, ICML-UNAM 10248).

**GUERRERO:** **Morro del Cerro Colorado** (171 specimens, stony coral, 1.5 m, 30/11/2010, MHN 005-4472; 36 specimens, stony coral, 3 m, 30/11/2010, MHN 005-4435; 19 specimens, stony coral, 2.7 m, 30/11/2010, MHN 005-4428; 40 specimens, rock, 30/11/2010, ICML-UNAM 10360; 6 specimens, algae, 30/11/2010, ICML-UNAM 10366; 3 specimens, rhodoliths, 30/11/2010, ICML-UNAM 10367; 16 specimens, sponge, 30/11/2010, ICML-UNAM 10363; 2 specimens, rock, 4.5 m, 23/11/2011, ICML-UNAM 10478; 4 specimens, rhodoliths, 4.5 m, 23/11/2011, ICML-UNAM 10479; 10 specimens, sponge, 4.5 m, 23/11/2011, ICML-UNAM 10480; 37 specimens, dead coral, 3 m, 31/05/2012, ICML-UNAM 10558; 27 specimens, rock, 5.5 m, 31/05/2012, ICML-UNAM 10559; 2 specimens, sponge, 5.5 m, 31/05/2012, ICML-UNAM 10560); **Carey** (5 specimens, rock, 4 m, 23/11/2011, ICML-UNAM 10496); **Zacatoso** (2 specimens, rock, 02/03/2009, ICML-UNAM 10174; 1 specimen, stony coral, 5.1 m, 18/02/2010, ICML-UNAM 10244; 20 specimens, stony coral, 5.1 m, 01/09/2010, ICML-UNAM 10256; 369 specimens, stony coral, 9.1 m, 01/12/2010, MHN 005-4481; 4 specimens, algae, 7.5 m, 01/12/2010, ICML-UNAM 10376; 3 specimens, sponge, 01/12/2010, ICML-UNAM 10372; 5 specimens, algae, 16.5 m, 02/12/2010, ICML-UNAM 10381; 2 specimens, rock, 25/11/2011, ICML-UNAM 10504; 30 specimens, rock, 9.1 m, 01/06/2012, ICML-UNAM 10574; 1 specimen, sponge, 9.1 m, 01/06/2012, ICML-UNAM 10575); **El Chato** (1 specimen, rock, 13.7 m, 04/03/2009, ICML-UNAM 10185); **Caleta de Chón** (1 specimen, rock, 03/03/2009, ICML-UNAM 10180; 26 specimens, stony coral, 4.6 m, 02/12/2010, MHN 005-4450; 39 specimens, stony coral, 6.1 m, 02/12/2010, MHN 005-4423; 52 specimens, stony coral, 7.6 m, 02/12/2010, MHN 005-4469; 3 specimens, rock, 02/12/2010, ICML-UNAM 10389; 2 specimens, rock, 5 m, 22/11/2011, ICML-UNAM 10473); **El Yunque** (15 specimens, rock, 5.5 m, 04/12/2010, ICML-UNAM 10405; 4 specimens, sponge, 5.5 m, 04/12/2010, ICML-UNAM 10406; 6 specimens, rock, 8 m, 24/11/2011, ICML-UNAM 10501); **Manzanillo** (5 specimens, stony coral, 3 m, 04/12/2010, MHN 005-4459; 1 specimen, rock, 04/12/2010, ICML-UNAM 10416; 10 specimens, rhodoliths, 5.3 m, 04/12/2010, ICML-UNAM 10411; 1 specimen, sponge, 6 m, 22/11/2011, ICML-UNAM 10465; 5 specimens, rock, 6 m, 22/11/2011, ICML-UNAM 10466; 16 specimens, dead coral, 6.1 m, 30/05/2012, ICML-UNAM 10545; 24 specimens, rock, 6.1 m, 30/05/2012, ICML-UNAM 10546; 4 specimens, sponge, 6.1 m, 30/05/2012, ICML-UNAM 10547); **Morros de Potosí** (5 specimens, rock, 06/03/2009, ICML-UNAM 10195; 1 specimen, sponge, 06/03/2009, ICML-UNAM 10196; 13 specimens, stony coral, 11.7 m, 01/09/2010, ICML-UNAM 10260; 57 specimens, stony coral, 6.1 m, 03/12/2010, MHN 005-4425; 49 specimens, stony coral, 7.6 m, 03/12/2010, MHN 005-4477; 124 specimens, stony coral, 10.7 m, 03/12/2010, MHN 005-4476; 14 specimens, rock, 03/12/2010, ICML-UNAM 10399; 4 specimens, algae, 12.2 m, 03/12/2010, ICML-UNAM 10390; 10 specimens, sponge, 03/12/2010, ICML-UNAM 10396); **Coral** (5 specimens, rock, 5 m, 23/11/2011, ICML-UNAM 10490); **Palmitas** (89 specimens, stony coral, 3.6 m, 03/09/2010, ICML-UNAM 10264; 16 specimens, rock, 6.4 m, 20/11/2011, ICML-UNAM 10449; 2 specimens, algae, 6.4 m, 20/11/2011, ICML-UNAM 10450; 4 specimens, sponge, 6.4 m, 20/11/2011, ICML-UNAM 10451); **El Ripial** (14 specimens, stony coral, 4.6 m, 03/09/2010, ICML-UNAM 10267; 6 specimens, 20/11/2011, ICML-UNAM 10460).

**OAXACA:** **Puerto Angelito** (5 specimens, rock, 10.7 m, 05/08/2007, MHN 005-4341; 23 specimens, rock, 10 m, 23/04/2012, ICML-UNAM 10507); **El Zapatito** (15 specimens, rock, 23/04/2009, ICML-UNAM 10221); **Punto de Presión** (3 specimens, rock, 26 m, 22/04/2009, ICML-UNAM 10205; 8 specimens, sponge, 26 m, 22/04/2009, ICML-UNAM 10206); **El Faro** (7 specimens, rock, 22.9 m, 22/04/2009, ICML-UNAM 10211; 13 specimens, rock, 23/04/2009, ICML-UNAM 10227; 23 specimens, rock, 13.5 m, 24/04/2012, ICML-UNAM 10513); **Mazunte** (4 specimens, stony coral, 4.6 m, 03/06/2010, MHN 005-4416); **Estacahuite** (2 specimens, stony coral, 7.6 m, 26/03/2010, MHN 005-4405; 6 specimens, stony coral, 8.8 m, 26/03/2010, MHN 005-4402; 3 specimens, stony coral, 9.7 m, 26/03/2010, MHN 005-4398; 7 specimens, stony coral, 10.3 m, 04/09/2010, ICML-UNAM 10268); **Boquilla** (7 specimens, stony coral, 6.3 m, 08/09/2010, ICML-UNAM 10277); **Salchi** (2 specimens, stony coral, 6.1 m, 26/03/2010, MHN 005-4412); **San Agustín** (2 specimens, stony coral, 6.4 m, 23/02/2010, MHN 005-4395); **Dos Hermanas** (1 specimen, rock, 08/08/2011, ICML-UNAM 10422); **La Entrega** (2 specimens, rock, 03/12/2008, MHN 005-4362; 8 specimens, stony coral, 5.3 m, 06/09/2010, ICML-UNAM 10272); **Isla Montosa** (1 specimen, stony coral, 3.6 m, 22/02/2010, MHN 005-4386; 1 specimen, stony coral, 5.8 m, 22/02/2010, MHN 005-4383); **Guerrilla** (10 specimens, rock, 4.9 m, 18/05/2012, ICML-UNAM 10535); **Copalita** (5 specimens, rock, 9.1 m, 18/05/2012, ICML-UNAM 10529).

##### *Ophiothela* Verrill, 1867

###### Ophiothela mirabilis

Verrill, 1867

http://species-id.net/wiki/Ophiothela_mirabilis

Figure 3A–F

####### Description.

**Figure 3.**
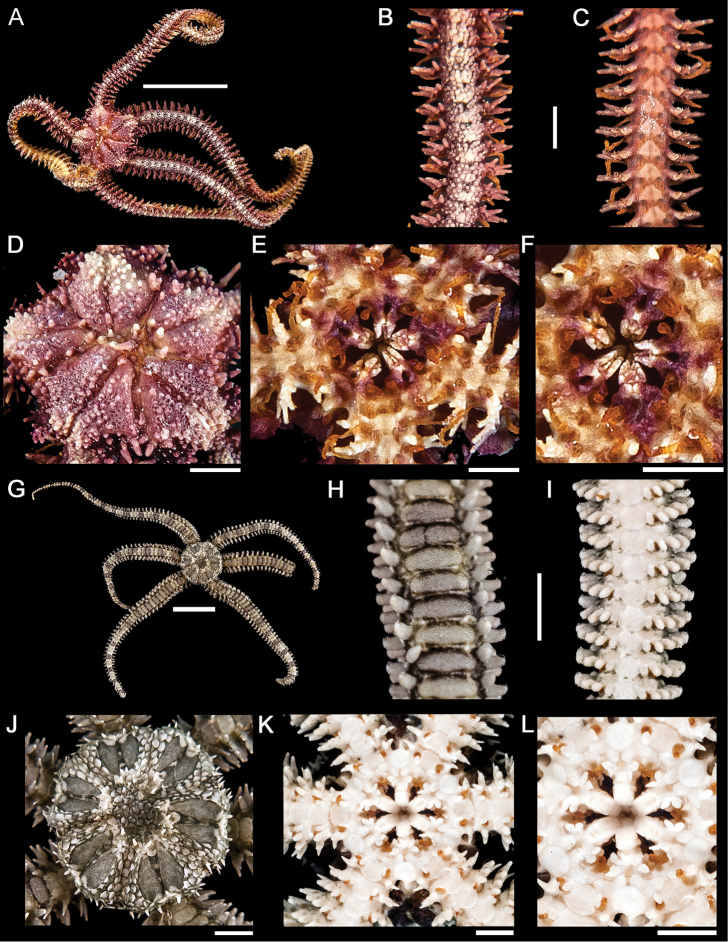
*Ophiothela mirabilis*. **A** dorsal view. Scale bar = 5 mm **B** dorsal view of the arm **C** ventral view of the arm **D** dorsal view of the disk **E** ventral view of the disk **F** jaw. Scale bar = 1 mm. *Ophiactis savignyi* **G** dorsal view. Scale bar = 5 mm **H** dorsal view of the arm **I** ventral view of the arm **J** dorsal view of the disk **K** ventral view of the disk **L** jaw. Scale bar = 1 mm.

Disk lobulated (dd = 1.3 to 4.3 mm) and totally covered by large, rounded and scattered grains of different sizes. Disk mostly covered by large radial shields which are in contact and covered by grains (Fig. 3D). The ventral side of the disk is covered by skin (Fig. 3E). Oral and adoral shields appear to be fused, forming a continuous ring; covered by skin. Oral papillae lacking. A cluster of rounded dental papillae; about 10–18 (Fig. 3F). Six rolled up arms. Dorsal arm plates concealed and covered by numerous rounded grains, there are naked spaces between plates (Fig. 3B). Ventral arm plates with rounded edges, separated by the lateral arm plates and covered with skin (Fig. 3C). Lateral arm plates well developed. Six arm spines provided with well-developed hooks at the tip; the third spine is the longest. Tentacle scales lacking. Color purplish-rosaceous; dorsal side of the disk mostly rosaceous with purplish arms (Fig. 3A), ventral side uniformly rosaceous (Fig. 3E).

####### Distribution.

Mexico, El Salvador, Costa Rica, Panama, Colombia, Brazil, Lesser Antilles (Verrill 1867, Neira and Cantera 2005, Alvarado et al. 2010, Hendler et al. 2012). In Mexico has been reported from the Gulf of California (Baja California Sur and Sinaloa), on the Pacific side of Baja California Sur, Nayarit, Jalisco, Michoacán, Guerrero and Oaxaca (Solís-Marín et al. 2005, Honey-Escandón et al. 2008, Granja-Fernández and López-Pérez 2011). Depth 6 to 61 m (Maluf 1988). In this study, *Ophiothela mirabilis* was collected on coral reefs from Jalisco, Michoacán, Guerrero and Oaxaca, between depths of 4 and 26 m.

####### Remarks.

*Ophiothela mirabilis* was collected in gorgonians and sponges, but was more numerous and conspicuous in the former. We observed that *Ophiothela mirabilis* had a great variety of colors (purple rosaceous, creamy, burgundy and yellow) which were correlated with the color of the gorgonians upon which the brittle stars were collected. This color matching between *Ophiothela* and gorgonians also has been reported by Clark AM (1976a). *Ophiothela mirabilis* is always attached to the gorgonians rolling up their arms and with the help of their hooked spines of the arms. Specimens were found in three forms: specimens with six well-developed arms, with six arms, of which three were not well-developed or with only three arms. This is correlated with asexual reproduction by fission where the disk splits in two parts and each split disk regenerates the missing part (Clark AM 1976a, Hendler et al. 2012). *Ophiothela mirabilis* has recently been found in emergent populations in the Atlantic and the Caribbean, where it may alter the appearance and ecology of the areas due to the high densities of the species (Hendler et al. 2012).

####### Collected material.

**JALISCO:** **La Pajarera** (55 specimens, gorgonian, 29/09/2010, ICML-UNAM 10300).

**MICHOACÁN:** **Isla Pájaros** (1 specimen, 28/09/2010, ICML-UNAM 10289).

**GUERRERO:** **Morro del Cerro Colorado** (35 specimens, gorgonian, 30/11/2010, ICML-UNAM 10361; 6 specimens, gorgonian, 4.5 m, 23/11/2011, ICML-UNAM 10481; 22 specimens, gorgonian, 5.5 m, 31/05/2012, ICML-UNAM 10561); **Carey** (3 specimens, gorgonian, 4 m, 23/11/2011, ICML-UNAM 10497); **Zacatoso** (7 specimens, gorgonian, 02/03/2009, ICML-UNAM 10175; 5 specimens, gorgonian, 9.1 m, 01/06/2012, ICML-UNAM 10576); **El Chato** (9 specimens, gorgonian, 13.7 m, 04/03/2009, ICML-UNAM 10186; 13 specimens, sponge, 04/03/2009, ICML-UNAM 10187); **Caleta de Chón** (4 specimens, gorgonian, 03/03/2009, ICML-UNAM 10181); **Manzanillo** (3 specimens, sponge, 05/03/2009, ICML-UNAM 10190; 2 specimens, gorgonian, 6 m, 22/11/2011, ICML-UNAM 10467); **Morros de Potosí** (24 specimens, gorgonian, 06/03/2009, ICML-UNAM 10197; 24 specimens, gorgonian, 06/03/2009, ICML-UNAM 10198; 159 specimens, gorgonian,, 03/12/2010, ICML-UNAM 10395); **Palmitas** (44 specimens, gorgonian, 6.4 m, 20/11/2011, ICML-UNAM 10452), **El Ripial** (46 specimens, gorgonian, 20/11/2011, ICML-UNAM 10461).

**OAXACA:** **El Zapatito** (3 specimens, sponge, 23/04/2009, ICML-UNAM 10222); **Punto de Presión** (30 specimens, sponge, 26 m, 22/04/2009, ICML-UNAM 10207); **El Faro** (14 specimens, gorgonian, 21.3 m, 22/04/2009, ICML-UNAM 10212); **La Mina** (10 specimens, gorgonian, 19/02/2009, MHN 005-4319); **San Agustín** (92 specimens, gorgonian, 16/04/2008, MHN 005-4345); **Órgano** (4 specimens, gorgonian, 08/08/2011, ICML-UNAM 10429); **Manzanilla** (1 specimen, gorgonian, 03/12/2008, MHN 005-4316); **Copalita** (26 specimens, gorgonian, 9.1 m, 18/05/2012, ICML-UNAM 10530).

#### Family Ophiactidae Matsumoto, 1915

##### *Ophiactis* Lütken, 1856

###### Ophiactis savignyi

(Müller & Troschel, 1842)

http://species-id.net/wiki/Ophiactis_savignyi\according to Granja–Fernández et al 2014

Figure 3G–L

####### Description.

Disk rounded (dd = 1.1 to 5 mm) and covered by large imbricated scales and short scattered spines over and at the edge of disk. Radial shields large, triangular and joining distally (Fig. 3J). Ventral interradius with scales and scattered spines (Fig. 3K). Oral shields diamond shaped with rounded edges. Adoral shields small and not meeting proximally. Two flattened oral papillae in each side of the jaw (Fig. 3L). Five or six arms. Dorsal arm plates wider than long with rounded edges (Fig. 3H). Ventral arm plates quadrangular with rounded edges. Five to six short, rugose and spinulose arm spines. Single, rounded, lanceolated tentacle scale (Fig. 3I). Color of the disk olive greenish-brown, with darker radial shields (Fig. 3G). Ventral side cream (Fig. 3K).

####### Distribution.

Cosmopolitan. Indo-Pacific, Eastern Pacific and both sides of the Atlantic. In the Eastern Pacific, the species has been recorded in the Hawaiian Islands, Mexico, Costa Rica, Panama, Colombia, Galapagos Islands and Peru (Clark HL 1915, Nielsen 1932, Maluf 1988, Hendler et al. 1995, Alvarado and Fernández 2005, Neira and Cantera 2005). In Mexico from the Gulf of California, on the Pacific side of Baja California and Baja California Sur, Nayarit, Marías Islands, Jalisco, Colima, Revillagigedo Islands, Michoacán, Guerrero and Oaxaca (Brusca 1980, Honey-Escandón et al. 2008). Commonly found in shallow water up to 518 m (Hendler et al. 1995). In this study, *Ophiactis savignyi* was collected on coral reefs from Nayarit, Jalisco, Colima, Michoacán, Guerrero and Oaxaca; between 2.3 to 26 m depth.

####### Remarks.

This small species is one of the most abundant on the coral reefs from the Mexican Pacific. Emson and Wilkie (1984) suggested that the sexual and asexual reproduction is important for the abundance and distribution of *Ophiactis savignyi*; small populations of this fissiparous brittle star may be increased by sexual reproduction. We found the species attached to live stony coral, dead coral, rock, algae, rhodoliths and sponges. Hendler et al. (1995) mentioned that this species is commonly found in all reef zones and substrata reported in this study, chiefly in sponges and algae. *Ophiactis savignyi* is a well-studied species; there are numerous works about its reproduction (e.g. Emson and Wilkie 1984, Hendler 1991, Chao and Tsai 1995, McGovern 2002), feeding (e.g. Boffi 1972, Emson and Mladenov 1992) and habitat (e.g. Boffi 1972, Sloan 1982, Hendler and Littman 1986, Neves et al. 2007). Other authors (Hendler et al. 1995, Pomory 2007) have reported that *Ophiactis savignyi* can have three oral papillae on each side of the mouth; most of the collected individuals in this study presented two oral papillae, with a few specimens presenting three.

####### Collected material.

**NAYARIT:** **Bahía Rabijuncos** (15 specimens, stony coral, 08/06/2011, ICML-UNAM 10433); **Las Monas** (18 specimens, stony coral, 17/06/2010, ICML-UNAM 10357).

**JALISCO:** **Isla Cocinas** (1 specimen, stony coral, 29/09/2010, ICML-UNAM 10327); **Pelícanos** (2 specimens, rock, 29/09/2010, ICML-UNAM 10318; 4 specimens, stony coral, 29/09/2010, ICML-UNAM 10319); **La Pajarera** (1 specimen, rhodolith, 29/09/2010, ICML-UNAM 10307); **La Palma** (1 specimen, stony coral, 29/09/2010, ICML-UNAM 10324); **La Virgencita** (18 specimens, stony coral, 30/09/2010, ICML-UNAM 10340).

**COLIMA:** **Carrizales** (2 specimens, 01/10/2010, ICML-UNAM 10349).

**MICHOACÁN:** **Faro de Bucerías** (4 specimens, 28/09/2010, ICML-UNAM 10296); **Isla Pájaros** (13 specimens, stony coral, 28/09/2010, ICML-UNAM 10290).

**GUERRERO:** **Morro del Cerro Colorado** (12 specimens, stony coral, 3 m, 30/11/2010, UMAR-MHN 005-4436; 4 specimens, sponge, 30/11/2010, ICML-UNAM 10364; 1 specimen, rock, 5.5 m, 31/05/2012, ICML-UNAM 10562; 11 specimens, dead coral, 3 m, 31/05/2012, ICML-UNAM 10563); **Coral** (1 specimen, rock, 5 m, 23/11/2011, ICML-UNAM 10491); **Zacatoso** (4 specimens, rock, 02/03/2009, ICML-UNAM 10176; 27 specimens, stony coral, 9.1 m, 01/12/2010, UMAR-MHN 005-4432; 16 specimens, algae, 2.3 m, 01/12/2010, ICML-UNAM 10377; 5 specimens, sponge, 02/12/2010, ICML-UNAM 10373; 8 specimens, algae, 5 m, 02/12/2010, ICML-UNAM 10382; 1 specimen, rock, 25/11/2011, ICML-UNAM 10505; 2 specimens, sponge, 9.1 m, 01/06/2012, ICML-UNAM 10577); **Caleta de Chón** (23 specimens, stony coral, 6.1 m, 02/12/2010, UMAR-MHN 005-4421; 10 specimens, stony coral, 4.6 m, 02/12/2010, UMAR-MHN 005-4452; 1 specimen, rock, 5 m, 22/11/2011, ICML-UNAM 10474); **Manzanillo** (38 specimens, sponge, 05/03/2009, ICML-UNAM 10191; 1 specimen, rock, 05/11/2009, ICML-UNAM 10238; 3 specimens, stony coral, 05/11/2009, ICML-UNAM 10239; 1 specimen, stony coral, 3 m, 04/12/2010, UMAR-MHN 005-4464; 2 specimens, rhodolith, 5.3 m, 04/12/2010, ICML-UNAM 10412; 4 specimens, rock, 6 m, 22/11/2011, ICML-UNAM 10468; 6 specimens, dead coral, 6.1 m, 30/05/2012, ICML-UNAM 10548; 6 specimens, sponge, 6.1 m, 30/05/2012, ICML-UNAM 10549); **Morros de Potosí** (1 specimen, rock, 06/03/2009, ICML-UNAM 10199; 3 specimens, sponge, 06/03/2009, ICML-UNAM 10200; 1 specimen, stony coral, 11.7 m, 01/09/2010, ICML-UNAM 10261; 26 specimens, stony coral, 6.1 m, 03/12/2010, MHN 005-4427; 9 specimens, stony coral, 10.7 m, 03/12/2010, MHN 005-4474; 16 specimens, stony coral, 7.6 m, 03/12/2010, MHN 005-4478; 3 specimens, algae, 12.2 m, 03/12/2010, ICML-UNAM 10391; 1 specimen, sponge, 03/12/2010, ICML-UNAM 10397; 1 specimen, rock, 03/12/2010, ICML-UNAM 10400); **Palmitas** (2 specimens, stony coral, 3.6 m, 03/09/2010, ICML-UNAM 10265; 1 specimen, algae, 6.4 m, 20/11/2011, ICML-UNAM 10453; 2 specimens, sponge, 6.4 m, 20/11/2011, ICML-UNAM 10454); **El Ripial** (1 specimen, 20/11/2011, ICML-UNAM 10462).

**OAXACA:** **Carrizalillo** (29 specimens, sponge, 4.2 m, 24/04/2012, ICML-UNAM 10520); **Punto de Presión** (13 specimens, sponge, 26 m, 22/04/2009, ICML-UNAM 10208); **El Faro** (3 specimens, rock, 22.8 m, 22/04/2009, ICML-UNAM 10213; 2 specimens, rock, 23/04/2009, ICML-UNAM 10228; 31 specimens, rock, 13.5 m, 24/04/2012, ICML-UNAM 10514); **Mazunte** (2 specimens, rock, 20/02/2009, MHN 005-4323); **Estacahuite** (17 specimens, stony coral, 7.6 m, 26/03/2010, MHN 005-4407; 2 specimens, stony coral, 8.8 m, 26/03/2010, MHN 005-4403; 7 specimens, stony coral, 9.7 m, 26/03/2010, MHN 005-4399); **Salchi** (2 specimens, stony coral, 6.1 m, 26/03/2010, MHN 005-4414); **San Agustín** (13 specimens, stony coral, 3.3 m, 23/02/2010, MHN 005-4394); **Maguey** (1 specimen, stony coral, 3.5 m, 06/09/2010, ICML-UNAM 10274); **La Entrega** (3 specimens, dead coral, 03/12/2008, MHN 005-4326); **Manzanilla** (3 specimens, dead coral, 3 m, 18/05/2012, ICML-UNAM 10523); **Isla Montosa** (1 specimens, stony coral, 5.8 m, 22/02/2010, MHN 005-4384; 1 specimen, stony coral, 6.1 m, 22/02/2010, MHN 005-4380).

###### Ophiactis simplex

(Le Conte, 1851)

http://species-id.net/wiki/Ophiactis_simplex

Figure 4A–F

####### Description.

**Figure 4.**
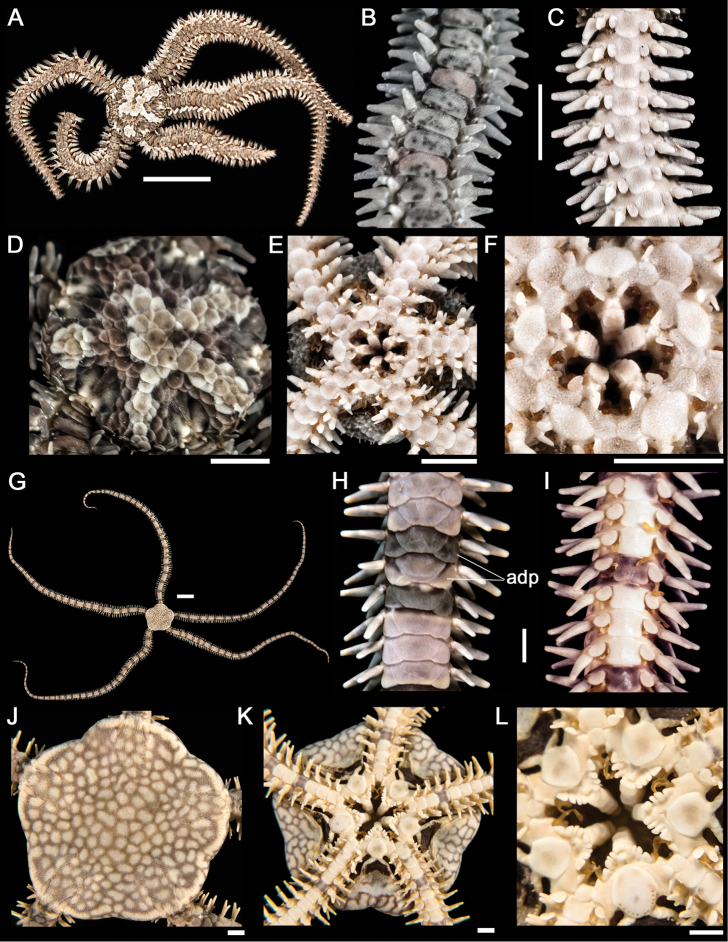
*Ophiactis simplex*. **A** dorsal view. Scale bar = 5 mm **B** dorsal view of the arm. **C** ventral view of the arm **D** dorsal view of the disk **E** ventral view of the disk **F** jaw. Scale bar = 1 mm. *Ophionereis annulata* **G** dorsal view. Scale bar = 5 mm **H** dorsal view of the arm (adp = accessory dorsal arm plates) **I** ventral view of the arm **J** dorsal view of the disk **K** ventral view of the disk **L** jaw. Scale bar = 1 mm.

Disk rounded (dd = 1.4 to 4.3 mm) covered by imbricating, rounded and large scales; the disk can bear small scattered spines. Primary plate conspicuous. Short spines at the edge of the disk. Radial shields small and widely separated by scales (Fig. 4D). Ventral side of the disk with spines and small imbricated scales (Fig. 4E). Oral shields oval, with a large madreporite shield evident. Adoral shields large about as wide as the oral shields, meeting within. One large oral papillae at each side of the jaw (Fig. 4F). Five arms. Dorsal arm plates wider than long with rounded edges (Fig. 4B). Ventral arm plates quadrangular with rounded edges. Lateral arm plates prominent. Four to five rounded and blunt arm spines, the lowest one shortest. A single, large and oval tentacle scale (Fig. 4C). Color of the dorsal side brown (Fig. 4A). Ventral side yellowish-cream, some ventral arm plates with darker bands (Fig. 4C, E).

####### Distribution.

USA (California), Mexico, Nicaragua, Costa Rica, Panama, Galapagos Islands and Peru (Nielsen 1932, Maluf 1988, Alvarado et al. 2010, Tirado et al. 2012). In Mexico, from the Gulf of California (Baja California Sur, Sonora, Sinaloa), on the Pacific side of Baja California and Baja California Sur, Nayarit, Marías Islands, Jalisco, Michoacán, Guerrero and Oaxaca (Solís-Marín et al. 2005, Honey-Escandón et al. 2008, Granja-Fernández and López-Pérez 2011, Ríos-Jara et al. 2013). In this study, *Ophiactis simplex* was collected on coral reefs from Nayarit, Jalisco, Colima, Michoacán, Guerrero and Oaxaca, from 1.5 to 22.3 m depth.

####### Remarks.

We collected *Ophiactis simplex* in the same substrata as *Ophiactis savignyi* (stony coral, dead coral, rocks, algae, rhodoliths and sponges). The results suggest that live stony coral is the preferred substrate for both species. Moreover, despite that both species inhabit sponges, *Ophiactis savignyi* was more numerous in this substrate than *Ophiactis simplex*. According to Lyman (1865), it is probable that larger organisms of *Ophiactis simplex* have five arms, while the smaller individuals have six. In this study, we only found five armed individuals of different sizes. Notwithstanding that both species of *Ophiactis* cohabit on the coral reefs of the Mexican Pacific, there are useful morphological characteristic to distinguish them apart: 1) small radial shields in *Ophiactis simplex* versus large and conspicuous radial shields in *Ophiactis savignyi*; 2) numerous spines on the dorsal side and on the margin of the disk in *Ophiactis savignyi* whilst *Ophiactis simplex* can have scattered spines on the disk; 3) a single and large oral papillae on each side of the jaw in *Ophiactis simplex* versus two or three small oral papillae in *Ophiactis savignyi*; 4) *Ophiactis simplex* have five arms and *Ophiactis savignyi* five or six; 5) smoother arms spines in *Ophiactis simplex*, and rugose-spinulose arm spines in *Ophiactis savignyi*. *Ophiactis simplex* is a new distribution record for the state of Colima.

####### Collected material.

**NAYARIT:** **Bahía Rabijuncos** (18 specimens, stony coral, 08/06/2011, ICML-UNAM 10434); **Las Pozas** (7 specimens, stony coral, 10/05/2011, ICML-UNAM 10420; 1 specimen, rock, 10/05/2011, ICML-UNAM 10421).

**JALISCO:** **Cuastecomatito** (2 specimens, 30/09/2010, ICML-UNAM 10338); **Isla Cocinas** (67 specimens, stony coral, 29/09/2010, ICML-UNAM 10328; 11 specimens, rock, 29/09/2010, ICML-UNAM 10329); **Pelícanos** (26 specimens, rock, 29/09/2010, ICML-UNAM 10320; 10 specimens, stony coral, 29/09/2010, ICML-UNAM 10321); **La Pajarera** (1 specimen, stony coral, 29/09/2010, ICML-UNAM 10308; 1 specimen, rock, 29/09/2010, ICML-UNAM 10309; 9 specimens, rhodolith, 29/09/2010, ICML-UNAM 10310); **La Palma** (8 specimens, stony coral, 29/09/2010, ICML-UNAM 10325; 27 specimens, rock, 29/09/2010, ICML-UNAM 10326); **La Virgencita** (93 specimens, stony coral, 30/09/2010, ICML-UNAM 10341).

**COLIMA:** **La Boquita** (1 specimen, stony coral, 2.5 m, 26/02/2010, ICML-UNAM 10253; 3 specimens, stony coral, 01/10/2010, ICML-UNAM 10355); **Carrizales** (1 specimen, stony coral, 01/10/2010, ICML-UNAM 10350; 5 specimens, sand, 01/10/2010, ICML-UNAM 10351).

**MICHOACÁN:** **Faro de Bucerías** (10 specimens, sand, 28/09/2010, ICML-UNAM 10297), **Isla Pájaros** (26 specimens, stony coral, 28/09/2010, ICML-UNAM 10291; 4 specimens, rock, 28/09/2010, ICML-UNAM 10292); **Morro Chino** (1 specimen, stony coral, 4.3 m, 23/02/2010, ICML-UNAM 10247).

**GUERRERO:** **Morro del Cerro Colorado** (2 specimens, stony coral, 1.5 m, 30/11/2010, MHN 005-4470; 29 specimens, stony coral, 3 m, 30/11/2010, MHN 005-4456; 4 specimens, stony coral, 2.7 m, 30/11/2010, MHN 005-4429; 2 specimens, algae, 30/11/2010, ICML-UNAM 10368; 5 specimens, rhodolith, 30/11/2010, ICML-UNAM 10369; 1 specimen, sponge, 30/11/2010, ICML-UNAM 10365; 2 specimens, stony coral, 4.5 m, 23/11/2011, ICML-UNAM 10486; 12 specimens, rock, 4.5 m, 23/11/2011, ICML-UNAM 10482; 153 specimens, dead coral, 3 m, 31/05/2012, ICML-UNAM 10564; 1 specimen, rock, 5.5 m, 31/05/2012, ICML-UNAM 10565); **Coral** (8 specimens, rock, 5 m, 23/11/2011, ICML-UNAM 10492; 5 specimens, rhodolith, 23/11/2011, ICML-UNAM 10487); **Zacatoso** (22 specimens, rock, 02/03/2009, ICML-UNAM 10177; 10 specimens, stony coral, 5.1 m, 01/09/2010, ICML-UNAM 10257; 8 specimens, algae, 7.5 m, 01/12/2010, ICML-UNAM 10378; 3 specimens, stony coral, 9.1 m, 01/12/2010, UMAR-MHN 005-4419; 2 specimens, sponge, 01/12/2010, ICML-UNAM 10374; 20 specimens, algae, 16.5 m, 02/12/2010, ICML-UNAM 10383); **Caleta de Chón** (14 specimens, stony coral, 4.6 m, 02/12/2010, MHN 005-4453; 22 specimens, stony coral, 6.1 m, 02/12/2010, MHN 005-4424; 8 specimens, stony coral, 7.6 m, 02/12/2010, MHN 005-4467; 2 specimens, algae, 4.5 m, 02/12/2010, ICML-UNAM 10386); **El Yunque** (1 specimen, 5 m, 04/12/2010, ICML-UNAM 10410); **Manzanillo** (1 specimen, sponge, 05/03/2009, ICML-UNAM 10192; 3 specimens, rock, 05/11/2009, ICML-UNAM 10240; 1 specimen, stony coral, 3 m, 04/12/2010, MHN 005-4465; 60 specimens, rhodolith, 5.3 m, 04/12/2010, ICML-UNAM 10413; 3 specimens, rock, 6 m, 22/11/2011, ICML-UNAM 10469; 113 specimens, dead coral, 6.1 m, 30/05/2012, ICML-UNAM 10550; 8 specimens, rock, 6.1 m, 30/05/12, ICML-UNAM 10551); **Morros de Potosí** (6 specimens, stony coral, 11.7 m, 01/09/2010, ICML-UNAM 10262; 5 specimens, stony coral, 6.1 m, 03/12/2010, MHN 005-4444; 24 specimens, stony coral, 7.6 m, 03/12/2010, MHN 005-4479; 35 specimens, stony coral, 10.7 m, 03/12/2010, MHN 005-4475; 1 specimen, rock, 16.7 m, 03/12/2010, ICML-UNAM 10401; 5 specimens, algae, 12.2 m, 03/12/2010, ICML-UNAM 10392; 1 specimen, sponge, 03/12/2010, ICML-UNAM 10398); **Palmitas** (1 specimen, sponge, 6.4 m, 20/11/2011, ICML-UNAM 10455).

**OAXACA:** **El Zapatito** (1 specimen, rock, 23/04/2009, ICML-UNAM 10223); **El Faro** (3 specimens, rock, 22.3 m, 22/04/2009, ICML-UNAM 10214; 1 specimen, rock, 13.5 m, 24/04/2012, ICML-UNAM 10515); **Mazunte** (9 specimens, stony coral, 4.5 m, 03/06/2010, MHN 005-4417); **Estacahuite** (3 specimens, stony coral, 6.4 m, 26/03/2010, MHN 005-4400; 25 specimens, stony coral, 7.6 m, 26/03/2010, MHN 005-4408; 6 specimens, stony coral, 8.8 m, 26/03/2010, MHN 005-4404;); **Salchi** (2 specimens, stony coral, 6.1 m, 26/03/2010, MHN 005-4379; 4 specimens, stony coral, 7.9 m, 26/03/2010, MHN 005-4410); **San Agustín** (5 specimens, stony coral, 6.4 m, 23/02/2010, MHN 005-4396); **La Entrega** (1 specimen, dead coral, 11/09/2007, MHN 005-4325); **Isla Montosa** (11 specimens, stony coral, 3.6 m, 22/02/2010, MHN 005-4391; 14 specimens, stony coral, 5.8 m, 26/03/2010, MHN 005-4384; 13 specimens, stony coral, 6.1 m, 26/03/2010, MHN 005-4380); **Guerrilla** (1 specimen, rock, 4.9 m, 18/05/2012, ICML-UNAM 10536).

#### Family Ophionereididae Ljungman, 1867

##### *Ophionereis* Lütken, 1859

###### Ophionereis annulata

(Le Conte, 1851)

http://species-id.net/wiki/Ophionereis_annulata

Figure 4G–L

####### Description.

Disk rounded (dd = 2.8 to 14.3 mm) and covered by small and imbricating scales. Central primary plate evident. Radial shields small, triangular and surrounded by large disk scales (Fig. 4J). Ventral interradius covered by imbricating scales, which are smaller than dorsal scales. Bursal slits large and with small genital papillae (Fig. 4K). Oral shields diamond-shape, longer than wide. The madreporite is evident. Adoral shields lanceolate, not meeting within. Four rounded and spaced oral papillae on each side of jaw. Single enlarged, triangular papillae found distally (Fig. 4L). Dorsal arm plates longer than wide. Accessory dorsal arm plates well developed (Fig. 4H). Ventral arm plates slightly longer than wide. Three arm spines with blunt tips. A single, flat and oval tentacle scale completely covering each tentacle pore (Fig. 4I). Disk light brown with closely spaced brown-purplish rounded spots. Arms with dark dorsal plates every fourth or fifth plate (Fig. 4G).

####### Distribution.

USA (California), Mexico, El Salvador, Costa Rica, Panama, Colombia, Ecuador and Galapagos Islands (Verrill 1867, Campbell 1921, Nielsen 1932, Ziesenhenne 1937, 1940, Neira and Cantera 2005, Honey-Escandón et al. 2008, Alvarado et al. 2010). In Mexico, from the Gulf of California (Baja California Sur, Sonora, Sinaloa), Nayarit, Jalisco, Revillagigedo Islands, Colima, Michoacán, Guerrero and Oaxaca (Solís-Marín et al. 2005, Honey-Escandón et al. 2008, Granja-Fernández et al. 2013). From 0 to 229 m depth (Maluf 1988). In this study, *Ophionereis annulata* was collected on coral reefs from Jalisco, Colima, Michoacán, Guerrero and Oaxaca, from 3.6 to 13.5 m depth.

####### Remarks.

Adult specimens of *Ophionereis annulata* were found buried in sand, while specimens found in live stony corals were juveniles. Maluf (1988) reports *Ophionereis annulata* inhabiting rock, algae and sponge in the Central Eastern Pacific, along with the substrata recorded in this study. This species is the only one in the Mexican Pacific having an association with a scale-worm (*Malmgrenia* cf. *variegata*); this association was found in localities from Jalisco, Guerrero and Oaxaca, Mexico (Granja-Fernández et al. 2013). This species is most closely related to *Ophionereis reticulata* (Say, 1825) from the east coast of America; although there are morphological characteristics that distinguish both species (Clark AM 1953). In the studied area, the color pattern of *Ophionereis annulata* displayed two variations: 1) the most common was of a creamy-yellowish-colored disk with brown spots or reticulations, while the arms were creamy and purplish but every fourth-fifth joint possessed a green-darker band; 2) the coloration of the disk was similar to the above but with smaller reticulations, the arms green in color with olive-green darker bands and blotches; the darker bands every fourth-fifth joint and, occupying one or two arm plates. These color patterns have been reported by different authors in other areas of the Eastern Pacific (Verrill 1867, Nielsen 1932, Ziesenhenne 1940, Caso 1951).

####### Collected material.

**JALISCO:** **La Pajarera** (2 specimens, 29/09/2010, ICML-UNAM 10301); **Pelícanos** (3 specimens, 29/09/2010, ICML-UNAM 10302); **Cuastecomatito** (1 specimen, sand, 30/09/2010, ICML-UNAM 10370).

**COLIMA:** **Punto B** (1 specimen, 01/10/2010, ICML-UNAM 10343).

**MICHOACÁN:** **Morro de Enmedio** (1 specimen, stony coral, 3.7 m, 24/02/2010, ICML-UNAM 10249).

**GUERRERO:** **Morro del Cerro Colorado** (5 specimens, sand, 4.5 m, 23/11/2011, ICML-UNAM 10483; 5 specimens, sand, 5.5 m, 31/05/2012, ICML-UNAM 10566); **Carey** (1 specimen, sand, 4 m, 23/11/2011, ICML-UNAM 10498); **Caleta de Chón** (7 specimens, sand, 03/03/2009, ICML-UNAM 10182); **El Yunque** (2 specimens, sand, 8 m, 24/11/2011, ICML-UNAM 10502); **Manzanillo** (5 specimens, sand, 05/11/2009, ICML-UNAM 10241; 2 specimens, sand, 6 m, 22/11/2011, ICML-UNAM 10470); **Morros de Potosí** (1 specimen, 06/03/2009, ICML-UNAM 10201).

**OAXACA:** **Puerto Angelito** (1 specimen, sand, 10.7 m, 05/08/2007, MHN 005-4375; 5 specimens, sand, 10 m, 23/04/2012, ICML-UNAM 10508); **El Faro** (3 specimens, sand, 22/04/2009, ICML-UNAM 10215; 2 specimens, sand, 23/04/2009, ICML-UNAM 10229; 7 specimens, sand, 13.5 m, 24/04/2012, ICML-UNAM 10516); **Mazunte** (1 specimen, sand, 17/04/2008, MHN 005-4299; 2 specimens, sand, 20/02/2009, ICML-UNAM 10171); **Estacahuite** (5 specimens, sand, 17/04/2008, MHN 005-4324; 3 specimens, sand, 19/02/2009, ICML-UNAM 10168); **La Mina** (4 specimens, sand, 17/04/2008, MHN 005-4334; 2 specimens, sand, 19/02/2009, MHN 005-4318); **Boquilla** (3 specimens, 19/09/2008, MHN 005-4336; 1 specimen, stony coral, 6.3 m, 08/09/2010, ICML-UNAM 10278); **Tijera** (2 specimens, sand, 16/04/2008, MHN 005-4291); **San Agustín** (1 specimen, sand, 6.4 m, 15/09/2007, MHN 005-4366); **Dos Hermanas** (3 specimens, sand, 08/08/2011, ICML-UNAM 10423); **Pomelo** (4 specimens, sand, 8.8 m, 21/10/2011, ICML-UNAM 10445); **Isla Cacaluta** (2 specimens, sand, 05/12/2008, MHN 005-4368); **Órgano** (1 specimen, sand, 08/08/2011, ICML-UNAM 10430); **Manzanilla** (2 specimens, sand, 03/12/2008, MHN 005-4301); **Isla Montosa** (3 specimens, stony coral, 3.6 m, 22/02/2010, MHN 005-4390); **Guerrilla** (2 specimens, sand, 4.9 m, 18/05/2012, ICML-UNAM 10537); **Copalita** (24 specimens, sand, 9.1 m, 18/05/2012, ICML-UNAM 10531).

#### Family Ophiocomidae Ljungman, 1867

##### *Ophiocoma* L. Agassiz, 1835

###### Ophiocoma aethiops

Lütken, 1859

http://species-id.net/wiki/Ophiocoma_aethiops

Figure 5A–F

####### Description.

**Figure 5.**
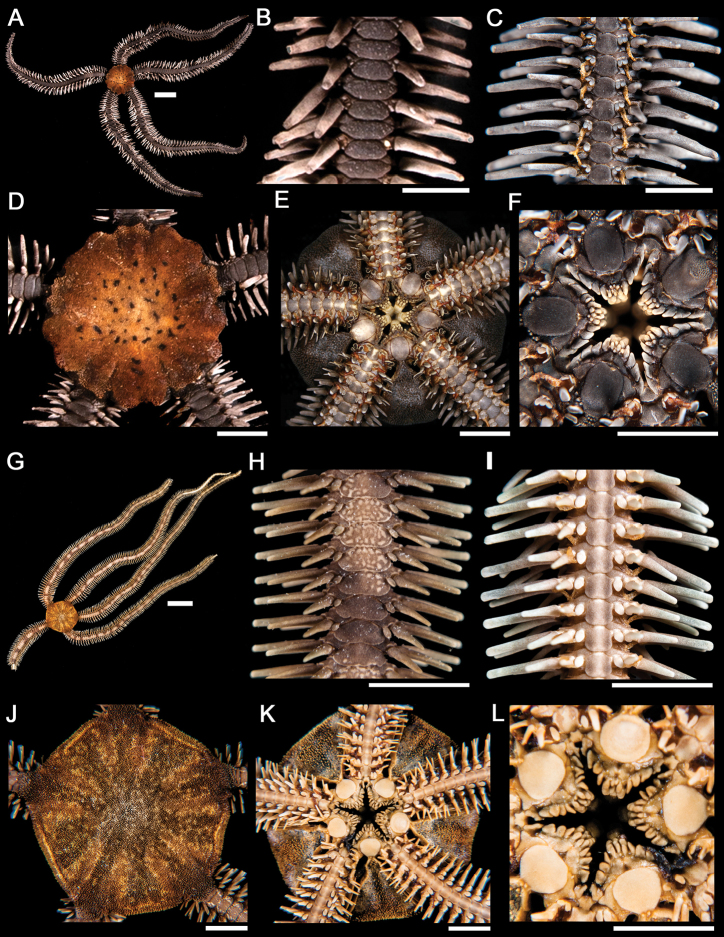
*Ophiocoma aethiops*. **A** dorsal view. Scale bar = 15 mm **B** dorsal view of the arm **C** ventral view of the arm **D** dorsal view of the disk **E** ventral view of the disk **F** jaw. Scale bar = 5 mm. *Ophiocoma alexandri* **G** dorsal view **H** dorsal view of the arm **I** ventral view of the arm. Scale bar = 15 mm **J** dorsal view of the disk **K** ventral view of the disk. **L** jaw. Scale bar = 5 mm.

Disk rounded (dd = 1.1 to 29.4 mm) and totally covered by fine granulation. The granulation covers the base of arms and the radial shields (Fig. 5D). The ventral side of the disc covered with scattered grains (Fig. 5E). Oral shields large, oblong; longer than wide. Adoral shields small, triangular and not meeting within. Cluster of rounded dental papillae on apex of jaw. Four oral papillae on each side of the jaw, the outer papillae being the largest. Four to five teeth (Fig. 5F). Dorsal arm plates wider than long, hexagonal shape (Fig. 5B). Ventral arm plates quadrangular with circular edges. Reduced lateral arm plates. Arm spines rounded, stout and slightly flattened. Arm spines, three and four, alternating. Two tentacle scales (Fig. 5C). Dorsal side dark brown-black in color. Disk with a brown large spot (Fig. 5A). Ventral arm plates and jaw with lighter dark color (Fig. 5K).

####### Distribution.

Lower California, Mexico, Guatemala, El Salvador, Honduras, Nicaragua, Costa Rica, Panama, Colombia, Peru, Galapagos Islands (Clark HL 1915, Hooker et al. 2005, Neira and Cantera 2005, Alvarado et al. 2010). In Mexico, from the Gulf of California (Baja California Sur, Sonora, Sinaloa), on the Pacific side of Baja California and Baja California Sur, Nayarit, Jalisco, Colima, Revillagigedo Islands, Michoacán, Guerrero and Oaxaca (Solís-Marín et al. 2005, Honey-Escandón et al. 2008, Granja-Fernández and López-Pérez 2011). From intertidal to 55 m depth (Maluf 1988). In this study, *Ophiocoma aethiops* was collected on coral reefs from Nayarit, Jalisco, Colima, Michoacán, Guerrero and Oaxaca, between depths of 2.1 to 26 m.

####### Remarks.

*Ophiocoma aethiops* is the largest brittle star on coral reefs from the Mexican Pacific. It is associated with live and dead stony coral, rocks, algae and rhodoliths. Juvenile organisms inhabit all the above mentioned substrata, meanwhile adults inhabit the interstitial spaces of live and dead stony corals and rocks. We observed that it is common to find juveniles attached to the dorsal side of the arms and disk of adults; an observation that has been previously recorded by Hendler et al. (1999) for Panama. *Ophiocoma aethiops* is a gonochoric species without sexual dimorphism (Benítez-Villalobos et al. 2012). On the Estacahuite reef of Oaxaca, females of *Ophiocoma aethiops* spawn from May to November, while male spawning occurs throughout the year, whereas the spawning of *Ophiocoma aethiops* in Panama occurs during November and December (Hendler 1979, Benítez-Villalobos et al. 2012). Besides the color pattern mentioned in the description of the species, we noted that some specimens can have a large white patch on the dorsal side of the disk, whereas the whole body of other specimens are black-brownish.

####### Collected material.

**NAYARIT:** **Islas Marietas** (1 specimen, stony coral, 2.1 m, 01/03/2010, ICML-UNAM 10254).

**JALISCO:** **Cuastecomatito** (5 specimens, rock, 30/09/2010, ICML-UNAM 10336); **Isla Cocinas** (9 specimens, 29/09/2010, ICML-UNAM 10304); **La Pajarera** (2 specimens, rock, 29/09/2010, ICML-UNAM 10311; 2 specimens, rhodolith, 29/09/2010, ICML-UNAM ); **La Virgencita** (1 specimen, stony coral, 30/09/2010, ICML-UNAM 10332); **Pelícanos** (2 specimens, 29/09/2010, ICML-UNAM 10315).

**COLIMA:** **Punto B** (3 specimens, 01/10/2010, ICML-UNAM 10344); **L’Recif** (1 specimen, 01/10/2010, ICML-UNAM 10346), **Carrizales** (1 specimen, stony coral, 01/10/2010, ICML-UNAM 10352).

**MICHOACÁN:** **Faro de Bucerías** (2 specimens, 28/09/2010, ICML-UNAM 10298); **Isla Pájaros** (9 specimens, stony coral, 28/09/2010, ICML-UNAM 10293; 1 specimen, dead coral, 28/09/2010, ICML-UNAM 10284; 3 specimens, rock, 28/09/2010, ICML-UNAM 10285); **Morro de Enmedio** (2 specimens, stony coral, 3.7 m, 24/02/2010, ICML-UNAM 10250).

**GUERRERO:** **Morro del Cerro Colorado** (1 specimen, rock, 4.5 m, 23/11/2011, ICML-UNAM 10484; 6 specimens, rock, 5.5 m, 31/05/2012, ICML-UNAM 10567); **Coral** (5 specimens, rock, 5 m, 23/11/2011, ICML-UNAM 10493; 3 specimens, rhodolith, 23/11/2011, ICML-UNAM 10488); **Carey** (1 specimen, rock, 4 m, 23/11/2011, ICML-UNAM 10499); **Zacatoso** (2 specimens, rock, 02/03/2009, ICML-UNAM 10178; 2 specimens, algae, 7.5 m, 01/12/2010, ICML-UNAM 10379; 1 specimen, algae, 16.5 m, 02/12/2010, ICML-UNAM 10384; 8 specimens, stony coral, 5.1 m, 01/09/2010, ICML-UNAM 10258; 1 specimen, rock, 9.1 m, 01/06/2012, ICML-UNAM 10578); **Caleta de Chón** (2 specimens, rock, 03/03/2009, ICML-UNAM 10183; 2 specimens, stony coral, 6.1 m, 02/12/2010, MHN 005-4420; 2 specimens, stony coral, 7.6 m, 26/03/2010, MHN 005-4466; 1 specimen, rock, 5 m, 22/11/2011, ICML-UNAM 10475); **El Yunque** (3 specimens, rock, 5.5 m, 04/12/2010, ICML-UNAM 10407); **Manzanillo** (1 specimen, rock, 05/11/2009, ICML-UNAM 10242; 2 specimens, rock, 05/03/2009, ICML-UNAM 10193; 2 specimens, rhodolith, 5.3 m, 04/12/2010, ICML-UNAM 10414; 4 specimens, rock, 6.1 m, 30/05/2012, ICML-UNAM 10552); **Morros de Potosí** (1 specimen, algae, 12.2 m, 03/12/2010, ICML-UNAM 10393); **Palmitas** (2 specimens, stony coral, 3.6 m, 03/09/2010, ICML-UNAM 10266; 21 specimens, rock, 6.4 m, 20/11/2011, ICML-UNAM 10456); **El Ripial** (1 specimen, 20/11/2011, ICML-UNAM 10463).

**OAXACA:** **Playa Coral** (1 specimen, stony coral, 2.3 m, 10/09/2010, ICML-UNAM 10281); **Puerto Angelito** (2 specimens, rock, 10 m, 23/04/2012, ICML-UNAM 10509); **El Zapatito** (2 specimens, rock, 23/04/2009, ICML-UNAM 10224); **Punto de Presión** (3 specimens, rock, 26 m, 23/04/2009, ICML-UNAM 10235); **El Faro** (2 specimens, rock, 22.3 m, 22/04/2009, ICML-UNAM 10216; 6 specimens, rock, 23/04/2009, ICML-UNAM 10230; 1 specimen, rock, 13.5 m, 24/04/2012, ICML-UNAM 10517); **Mazunte** (8 specimens, rock, 17/04/2008, MHN 005-4303; 2 specimens, rock, 20/02/2009, ICML-UNAM 10172); **Estacahuite** (7 specimens, rock, 17/04/2008, MHN 005-4313; 3 specimens, stony coral, 7.6 m, 26/03/2010, MHN 005-4406; 1 specimen, stony coral, 10.3 m, 04/09/2010, ICML-UNAM 10269); **La Mina** (8 specimens, rock, 17/04/2008, MHN 005-4293); **Boquilla** (1 specimen, stony coral, 6.3 m, 08/09/2010, ICML-UNAM 10279); **Tijera** (1 specimen, rock, 16/04/2008, MHN 005-4290); **San Agustín** (1 specimen, rock, 11/09/2007, MHN 005-4288; 5 specimens, rock, 15/09/2007, MHN 005-4360); **Riscalillo** (8 specimens, rock, 04/12/2008, MHN 005-4302); **Jicaral** (1 specimen, rock, 11/09/2007, MHN 005-4358); **Dos Hermanas** (7 specimens, rock, 08/08/2011, ICML-UNAM 10424); **Harrys** (7 specimens, rock, 9.1 m, 21/10/2011, ICML-UNAM 10436); **Pomelo** (3 specimens, rock, 8.9 m, 21/10/2011, ICML-UNAM 10446); **Copal** (3 specimens, rock, 9.1 m, 21/10/2011, ICML-UNAM 10441); **Maguey** (3 specimens, stony coral, 3.5 m, 06/09/2010, ICML-UNAM 10275); **Manzanilla** (11 specimens, rock, 03/12/2008, MHN 005-4308; 2 specimens, dead coral, 3 m, 18/05/2012, ICML-UNAM 10524); **Isla Montosa** (6 specimens, stony coral, 2.7 m, 22/02/2010, MHN 005-4382; 3 specimens, stony coral, 3.6 m, 22/02/2010, MHN 005-4392); **Guerrilla** (4 specimens, rock, 4.9 m, 18/05/2012, ICML-UNAM 10538); **Copalita** (3 specimens, rock, 9.1 m, 18/05/2012, ICML-UNAM 10532).

###### Ophiocoma alexandri

Lyman, 1860

http://species-id.net/wiki/Ophiocoma_alexandri

Figure 5G–L

####### Description.

Disk rounded (dd = 1.3 to 25.2 mm), covered by elongated grains. Radial shields totally covered by grains as well as the base of the arms (Fig. 5J). Ventral side of the disk covered with similar grains, but less numerous (Fig. 5K). Oral shields round. Adoral shields small and not meeting within. Four to five oral papillae on each side of the jaw, being the outer one lager. Cluster of rounded dental papillae on apex of jaw. Three of four teeth (Fig. 5L). Dorsal arm plates oval heart-shaped, wider than long (Fig. 5H). Ventral arm plates quadrangular with circular edges, wider than long (Fig. 5I). Reduced lateral arm plates. Five to seven long, stout, blunt arm spines. On first two to four joints, two lanceolated tentacle scales occur, and only one for remainder of the arm (Fig. 5K). Disk brown in color; dorsal arm plates light brown in color, some plates banded (Fig. 5G). Ventral arm plates with a brown longitudinal stripe along the arm (Fig. 5I).

####### Distribution.

USA, Mexico, Guatemala, El Salvador, Honduras, Nicaragua, Costa Rica, Panama, Colombia, Galapagos Islands (Ziesenhenne 1937, Neira and Cantera 2005, Alvarado et al. 2010). In Mexico, from the Gulf of California (Baja California Sur, Sonora, Sinaloa), on the Pacific side of Baja California and Baja California Sur, Nayarit, Jalisco, Colima, Revillagigedo Islands, Michoacán, Guerrero and Oaxaca (Solís-Marín et al. 2005, Honey-Escandón et al. 2008, Granja-Fernández and López-Pérez 2011). From intertidal to 70 m depth (Maluf 1988). In this study, *Ophiocoma alexandri* was collected on coral reefs from Nayarit, Jalisco, Colima, Michoacán, Guerrero and Oaxaca; 2.3 to 26 m depth.

####### Remarks.

*Ophiocoma aethiops* and *Ophiocoma alexandri* are the largest species on the studied coral reefs. While *Ophiocoma aethiops* has a larger disk, *Ophiocoma alexandri* has larger arms. Both species can be easily identified in the field with *Ophiocoma aethiops* being more robust and darker color pattern, whereas *Ophiocoma alexandri* presents a characteristic longitudinal line along the ventral arm plates. We observed different color patterns on *Ophiocoma alexandri* from dark brown, light brown, yellowish and green and in all cases some dorsal arm plates were banded and the darker longitudinal stripes on the ventral plates were evident. The wide range of color patterns observed in *Ophiocoma* can be explained by the diurnal color change of the species that involves a replacement of brown shades by greys and black in late afternoon. The observed banded pattern may provide camouflage from predators (fish) (Hendler 1984). *Ophiocoma alexandri* and *Ophiocoma aethiops* were collected in the same substrata (live and dead stony coral, rocks, algae and rhodoliths), and the adults of both species inhabit live and dead stony coral and rocks. Similar to *Ophiocoma aethiops*, *Ophiocoma alexandri* is a gonochoric species, without sexual dimorphism (Benítez-Villalobos et al. 2012). Female spawning in Estacahuite reef (Oaxaca) occurs from May to December while male spawning occurs throughout the year. The spawning of *Ophiocoma alexandri* in Panama occurs during November and December (Hendler 1979, Benítez-Villalobos et al. 2012).

####### Collected material.

**NAYARIT:** **Las Monas** (1 specimen, rock, 09/05/2011, ICML-UNAM 10419; 4 specimens, stony corals, 17/06/2010, ICML-UNAM 10358); **Bahía Rabijuncos** (1 specimen, stony coral, 08/06/2011, ICML-UNAM 10435).

**JALISCO:** **Cuastecomatito** (19 specimens, rock, 30/09/2010, ICML-UNAM 10337); **Isla Cocinas** (1 specimen, stony coral, 29/09/2010, ICML-UNAM 10330; 1 specimen, rock, 29/09/2010, ICML-UNAM 10331); **La Pajarera** (15 specimens, stony coral, 29/09/2010, ICML-UNAM 10312; 13 specimens, rock, 29/09/2010, ICML-UNAM 10313; 1 specimen, rhodolith, 29/09/2010, ICML-UNAM 10314); **La Palma** (3 specimens, rock, 29/09/2010, ICML-UNAM 10333); **La Virgencita** (14 specimens, stony coral, 30/09/2010, ICML-UNAM 10342); **Pelícanos** (17 specimens, 29/09/2010, ICML-UNAM 10303).

**COLIMA:** **Punto B** (3 specimens, 01/10/2010, ICML-UNAM 10345); **Carrizales** (3 specimens, 01/10/2010, ICML-UNAM 10353); **L’Recif** (2 specimens, 01/10/2010, ICML-UNAM 10347).

**MICHOACÁN:** **Faro de Bucerías** (1 specimen, 28/09/2010, ICML-UNAM 10299); **Isla Pájaros** (4 specimens, stony coral, 28/09/2010, ICML-UNAM 10294; 6 specimens, dead coral, 28/09/2010, ICML-UNAM 10286; 8 specimens, rock, 28/09/2010, ICML-UNAM 10287); **Morro de Enmedio** (4 specimens, stony coral, 3.7 m, 24/02/2010, ICML-UNAM 10251).

**GUERRERO:** **Morro del Cerro Colorado** (3 specimens, stony coral, 3 m 30/11/2010, MHN 005-4434; 1 specimen, rock, 30/11/2010, ICML-UNAM 10362; 8 specimens, rock, 4.5 m, 23/11/2011, ICML-UNAM 10485; 7 specimens, dead coral, 3.1 m, 31/05/2012, ICML-UNAM 10568; 6 specimens, rock, 5.5 m, 31/05/2012, ICML-UNAM 10569); **Coral** (9 specimens, rock, 5 m, 23/11/2011, ICML-UNAM 10494); **Zacatoso** (7 specimens, rock, 02/03/2009, ICML-UNAM 10179; 1 specimen, stony coral, 9.1 m, 01/12/2010, MHN 005-4445; 1 specimen, algae, 2.3 m, 01/12/2010, ICML-UNAM 10380; 1 specimen, stony coral, 5.1 m, 18/02/2010, ICML-UNAM 10243; 5 specimens, stony coral, 5.1 m, 01/09/2010, ICML-UNAM 10259; 9 specimens, rock, 9.1 m, 01/06/2012, ICML-UNAM 10579); **El Chato** (2 specimens, rock, 13.7 m, 04/03/2009, ICML-UNAM 10188); **Caleta de Chón ** (8 specimens, rock, 03/03/2009, ICML-UNAM 10184; 1 specimen, algae, 4.5 m, 02/12/2010, ICML-UNAM 10387; 4 specimens, rock, 5 m, 22/11/2011, ICML-UNAM 10476); **El Yunque** (4 specimens, rock, 5.5 m, 04/12/2010, ICML-UNAM 10408; 3 specimens, rock, 8 m, 24/11/2011, ICML-UNAM 10503); **Manzanillo** (9 specimens, rock, 05/03/2009, ICML-UNAM 10194; 2 specimens, rock, 04/12/2010, ICML-UNAM 10417; 2 specimens, rhodolith, 5.3 m, 04/12/2010, ICML-UNAM 10415; 7 specimens, rock, 6 m, 22/11/2011, ICML-UNAM 10471; 52 specimens, dead coral, 6.1 m, 30/05/2012, ICML-UNAM 10553; 15 specimens, rock, 6.1 m, 30/05/2012, ICML-UNAM 10554); **Morros de Potosí** (5 specimens, rock, 06/03/2009, ICML-UNAM 10202; 2 specimens, stony coral, 11.7 m, 01/09/2010, ICML-UNAM 10263; 1 specimen, stony coral, 6.1 m, 03/12/2010, MHN 005-4442; 1 specimen, rock, 03/12/2010, ICML-UNAM 10402; 2 specimens, algae, 12.2 m, 03/12/2010, ICML-UNAM 10394); **Palmitas** (18 specimens, rock, 6.4 m, 20/11/2011, ICML-UNAM 10457); **El Ripial** (5 specimens, 20/11/2011, ICML-UNAM 10464).

**OAXACA:** **Playa Coral** (1 specimen, stony coral, 2.3 m, 10/09/2010, ICML-UNAM 10282); **Carrizalillo** (1 specimen, rock, 4.2 m, 24/04/2012, ICML-UNAM 10521); **Puerto Angelito** (4 specimens, rock, 10.7 m, 05/08/2007, MHN 005-4344; 10 specimens, rock, 10 m, 23/04/2012, ICML-UNAM 10510); **El Zapatito** (1 specimen, rock, 23/04/2009, ICML-UNAM 10225); **Punto de Presión** (2 specimens, rock, 26 m, 23/04/2009, ICML-UNAM 10236); **El Faro** (3 specimens, rock, 22.9 m, 22/04/2009, ICML-UNAM 10217; 1 specimen, hydrozoan, 21.3 m, 22/04/2009, ICML-UNAM 10218; 39 specimens, rock, 23/04/2009, ICML-UNAM 10231; 7 specimens, rock, 13.5 m, 24/04/2012, ICML-UNAM 10518); **Mazunte** (4 specimens, rock, 17/04/2008, MHN 005-4300; 6 specimens, rock, 20/02/2009, ICML-UNAM 10173; 2 specimens, stony coral, 4.6 m, 03/06/2010, MHN 005-4415); **Estacahuite** (9 specimens, rock, 21/09/2007, MHN 005-4370; 4 specimens, rock, 17/04/2008, MHN 005-4367; 4 specimens, rock, 19/02/2009, ICML-UNAM 10169; 1 specimen, stony coral, 7.6 m, 26/03/2010, MHN 005-4406; 1 specimen, stony coral, 9.7 m, 26/03/2010, MHN 005-4401; 4 specimens, stony coral, 10.3 m, 04/09/2010, ICML-UNAM 10270); **La Mina** (9 specimens, rock, 17/04/2008, MHN 005-4305; 8 specimens, rock, 19/02/2009, ICML-UNAM 10170); **Tijera** (6 specimens, rock, 16/04/2008, MHN 005-4306); **Boquilla** (8 specimens, rock, 21/09/2007, MHN 005-4310; 3 specimens, stony coral, 6.3 m, 08/09/2010, ICML-UNAM 10280); **Salchi** (13 specimens, stony coral, 6.1 m, 26/03/2010, MHN 005-4411; 6 specimens, stony coral, 7.9 m, 26/03/2010, MHN 005-4409); **San Agustín** (1 specimen, rock, 11/09/2007, MHN 005-4288; 2 specimens, rock, 15/09/2007, MHN 005-4361; 1 specimen, stony coral, 3.3 m, 23/02/2010, MHN 005-4397; 2 specimens, stony coral, 5.8 m, 23/02/2010, MHN 005-4393); **Riscalillo** (26 specimens, rock, 04/12/2008, MHN 005-4311); **Jicaral** (1 specimen, rock, 11/09/2007, MHN 005-4350); **Dos Hermanas** (8 specimens, rock, 08/08/2011, ICML-UNAM 10425); **Harrys** (10 specimens, rock, 9.1 m, 21/10/2011, ICML-UNAM 10437); **Pomelo** (12 specimens, rock, 8.8, 21/10/2011, ICML-UNAM 10447); **Copal** (10 specimens, rock, 9.1 m, 21/10/2011, ICML-UNAM 10442); **Isla Cacaluta** (2 specimens, 11/09/2007, MHN 005-4337; 9 specimens, stony coral, 6.9 m, 06/09/2010, ICML-UNAM 10276); **Órgano** (5 specimens, rock, 08/08/2011, ICML-UNAM 10431); **La Entrega** (2 specimens, rock, 03/12/2008, MHN 005-4228; 8 specimens, stony coral, 5.3 m, 06/09/2010, ICML-UNAM 10273); **Manzanilla** (15 specimens, rock, 03/12/2008, MHN 005-4309; 2 specimens, dead coral, 3 m, 18/05/2012, ICML-UNAM 10525); **Isla Montosa** (21 specimens, stony coral, 3.6 m, 22/02/2010, MHN 005-4388; 1 specimen, stony coral, 5.8 m, 22/02/2010, MHN 005-4384) **Guerrilla** (10 specimens, rock, 4.9 m, 18/05/2012, ICML-UNAM 10539).

#### Family Ophiodermatidae Ljungman, 1867

##### *Ophioderma* Müller & Troschel, 1840

###### Ophioderma panamensis

Lütken, 1859

http://species-id.net/wiki/Ophioderma_panamensis

Figure 6A–F

####### Description.

**Figure 6.**
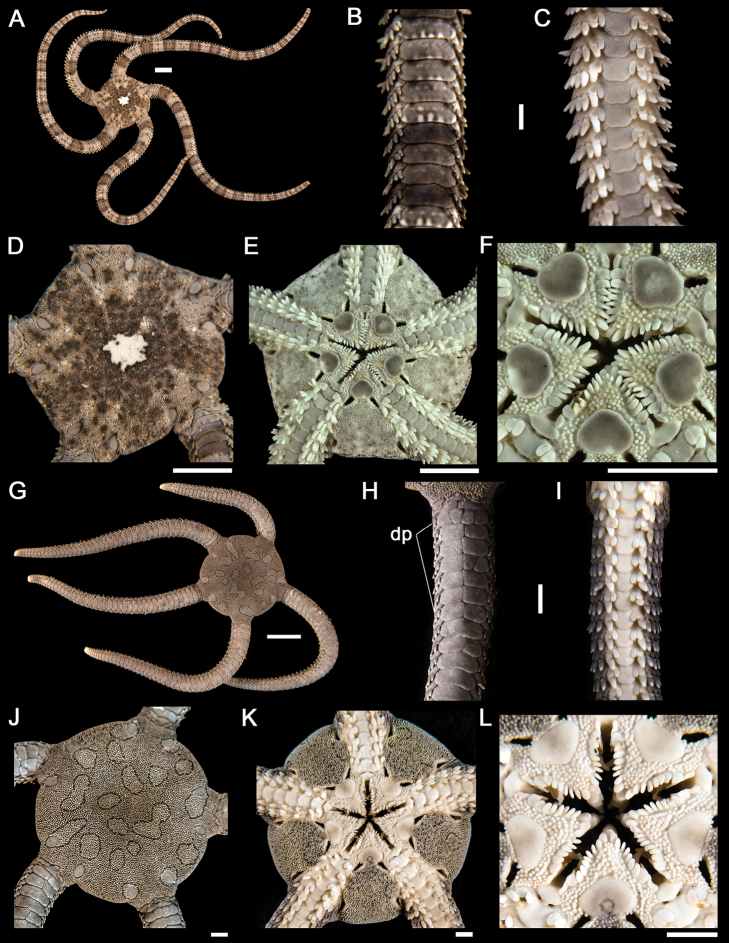
*Ophioderma panamensis*. **A** dorsal view **B** dorsal view of the arm **C** ventral view of the arm **D** dorsal view of the disk **E** ventral view of the disk **F** jaw. Scale bar = 5 mm. *Ophioderma teres* juvenile **G** dorsal view. Scale bar = 5 mm **H** dorsal view of the arm (dp = divided dorsal arm plates) **I** ventral view of the arm **J** dorsal view of the disk **K** ventral view of the disk **L** jaw. Scale bar = 1 mm.

Disk pentagonal (dd = 4.7 to 18.1 mm); dorsal and ventral side covered by fine, closely and rounded granulation. Radial shields naked, small and oval (Fig. 6D, E). Oral shields large and oval; wider than long. Adoral shields covered by granules. The madreporite is evident. Eight to ten oral papillae on each side of the jaw (Fig. 6F). Dorsal arm plates overlapping and rectangular with rounded edges; wider than long (Fig. 6B). Ventral arm plates oval and slightly overlapping; wider than long. Reduced lateral arm plates. Ten to 11 short and blunt arm spines; all spines are closely equal in size except the lowest which is longer. Two lanceolated tentacle scales (Fig. 6C). Four bursal slits per interradius (Fig. 6E). Disk brownish (Fig. 6A, D). Dorsal arm plates brown with dark and light bands (Fig. 6B).

####### Distribution.

USA (California), Mexico, El Salvador, Nicaragua, Costa Rica, Panama, Peru, Colombia and Galapagos Islands (Clark HL 1940, Hooker et al. 2005, Neira and Cantera 2005, Alvarado et al. 2010). In Mexico, from the Gulf of California (Baja California Sur, Sonora, Sinaloa), on the Pacific side of Baja California and Baja California Sur, Jalisco, Revillagigedo Islands, Guerrero and Oaxaca (Solís-Marín et al. 2005, Honey-Escandón et al. 2008, Granja-Fernández and López-Pérez 2011, 2012). From intertidal to 40 m depth (Austin and Hadfield 1980). In this study, *Ophioderma panamensis* was collected on coral reefs from Guerrero and Oaxaca, 4.9 to 9.1 m depth.

####### Remarks.

*Ophioderma panamensis* was collected under rocks and on sand. Maluf (1988) reported that this species may inhabit rocks, corals and algae. Adults and juveniles were found cohabiting together. Juveniles curled their arms over the dorsal side of the disk which is white in most individuals. This behavior camouflages juveniles on the white sand under the rocks. There are studies that report a wide variety of color patterns in *Ophioderma panamensis* (Ives 1889, Nielsen 1932, Ziesenhenne 1955) but, we only found two variations in body colors: brown or grey. In addition, some specimens had white spots on the disk and all specimens presented bands on the dorsal side of the arms. Although some authors report a high incidence of subdivided dorsal arm plates (Ives 1889, Nielsen 1932, Ziesenhenne 1955), we found only a few specimens with divided (in two segments) dorsal arm plates. *Ophioderma panamensis* was the most common ophiodermatid on the coral reefs from the Mexican Pacific.

####### Collected material.

**GUERRERO:** **Morro del Cerro Colorado** (1 specimen, rock, 5.5 m, 31/05/2012, ICML-UNAM 10570); **Zacatoso** (3 specimens, rock, 9.1 m, 01/06/2012, ICML-UNAM 10580); **El Yunque** (3 specimens, rock, 5.5 m, 04/12/2010, ICML-UNAM 10409); **Manzanillo** (1 specimen, rock, 6.1 m, 30/05/2012, ICML-UNAM 10555); **Morros de Potosí** (2 specimens, rock, 06/03/2009, ICML-UNAM 10203); **Palmitas** (5 specimens, rock, 6.4 m, 20/11/2011, ICML-UNAM 10458).

**OAXACA:** **El Zapatito** (1 specimen, rock, 23/04/2009, ICML-UNAM 10226); **Estacahuite** (1 specimen, rock, 17/04/2008, MHN 005-4354); **La Mina** (1 specimen, rock, 17/04/2008, MHN 005-4343); **Boquilla** (7 specimens, sand, 02/11/2007, MHN 005-4397); **Tijera** (1 specimen, 23/11/2007, MHN 005-401); **Dos Hermanas** (1 specimen, rock, 08/08/2011, ICML-UNAM 10426); **Harrys** (4 specimens, rock, 9.1 m, 21/10/2011, ICML-UNAM 10438); **Guerrilla** (2 specimens, rock, 4.9 m, 18/05/2012, ICML-UNAM 10540).

###### Ophioderma teres

(Lyman, 1860)

http://species-id.net/wiki/Ophioderma_teres

Figure 6G–L

####### Description.

Disk pentagonal (dd = 5.3 to 12.2 mm). Dorsal and ventral sides of the disk covered by fine and rounded granulation. Radial shields naked and oval (Fig. 6J, K). Oral shields heart-shaped, small and with three rounded lobes. Adoral shields covered by granulation. Madreporite evident. Eight to nine oral papillae on each side of the jaw (Fig. 6L). Dorsal arm plates wider than long and divided in several irregular pieces (two to four) (Fig. 6H). Ventral arm plates quadrangular and rounded. Reduced lateral arm plates. Six to seven short arm spines, all spines are closely equal in size, except the lowest which is longer. Two tentacle scales (Fig. 6I). Four bursal slits per interradius (Fig. 6K). Dorsal side uniformly chocolate-brown in color, with irregular black rings on the disk (Fig. 6G, J). Ventral side yellowish-cream in color (Fig. 6K).

####### Distribution.

Mexico, El Salvador, Nicaragua, Costa Rica, Panama, Ecuador, Colombia and Galapagos Islands (Ziesenhenne 1955, Neira and Cantera 2005, Alvarado et al. 2010). In Mexico, from the Gulf of California (Baja California Sur, Sonora, Sinaloa), in the Pacific side of Baja California and Baja California Sur, Nayarit, Jalisco, Guerrero and Oaxaca (Solís-Marín et al. 2005, Honey-Escandón et al. 2008). From intertidal to 46 m depth (Maluf 1988). In this study, *Ophioderma teres* was collected on coral reefs from Guerrero and Oaxaca; 9.1 to 10.7 m depth.

####### Remarks.

All the collected material of *Ophioderma teres* corresponded to juvenile specimens according to the juvenile species description by Lyman (1860). Adults are distinguished from juveniles by having grain-covered radial shields, a large number of subdivided dorsal plates in the arms (up to five), nine arm spines, and body uniformly dark-brown in color (Lyman 1860, Ziesenhenne 1955). Many juvenile specimens have been collected in the Gulf of California, Pacific side of Mexico, Panama, Ecuador and Galapagos Islands (Lyman 1860, Caso 1951, Ziesenhenne 1955). Several authors suggested that adults of *Ophioderma teres* might be confused with *Ophioderma panamensis* and proposed some characteristics to distinguish them: 1) shorter and rounded arms in *Ophioderma teres* versus larger and flattened arms in *Ophioderma panamensis*; 2) nine arm spines in *Ophioderma teres* and 11 in *Ophioderma panamensis*; 3) dorsal arm plates divided into three-five plates in *Ophioderma teres*, meanwhile some individuals of *Ophioderma panamensis* could have up to two; 4) radial shields covered in *Ophioderma teres* but naked in *Ophioderma panamensis*; 5) uniform brown color without banded arms in *Ophioderma teres* but colored and banded arms in *Ophioderma panamensis* (Lyman 1860, Verrill 1867, Nielsen 1932, Clark HL 1940, Ziesenhenne 1955). Similar to *Ophioderma panamensis*, *Ophioderma teres* was found on rocks and in sand.

####### Collected material.

**GUERRERO:** **Zacatoso** (3 specimens, rock, 9.1 m, 01/06/2012, ICML-UNAM 10581).

**OAXACA:** **Puerto Angelito** (1 specimen, rock, 10.7 m, 05/08/2007, MHN 005-4330); **El Faro** (2 specimens, rock, 23/04/2009, ICML-UNAM 10232); **Estacahuite** (4 specimens, rock, 18/09/2008, MHN 005-4332; 1 specimen, stony coral, 10.3 m, 04/09/2010, ICML-UNAM 10271); **La Mina** (2 specimens, rock, 17/04/2008, MHN 005-4331); **Boquilla** (1 specimen, 02/11/2007, MHN 005-4399); **Dos Hermanas** (3 specimens, rock, 08/08/2011, ICML-UNAM 10427); **Harrys** (1 specimen, sand, 9.1 m, 21/10/2011, ICML-UNAM 10439); **Copal** (1 specimen, sand, 9.1 m, 21/10/2011, ICML-UNAM 10443).

###### *Ophioderma* sp. 1

Figure 7A–F

**Figure 7.**
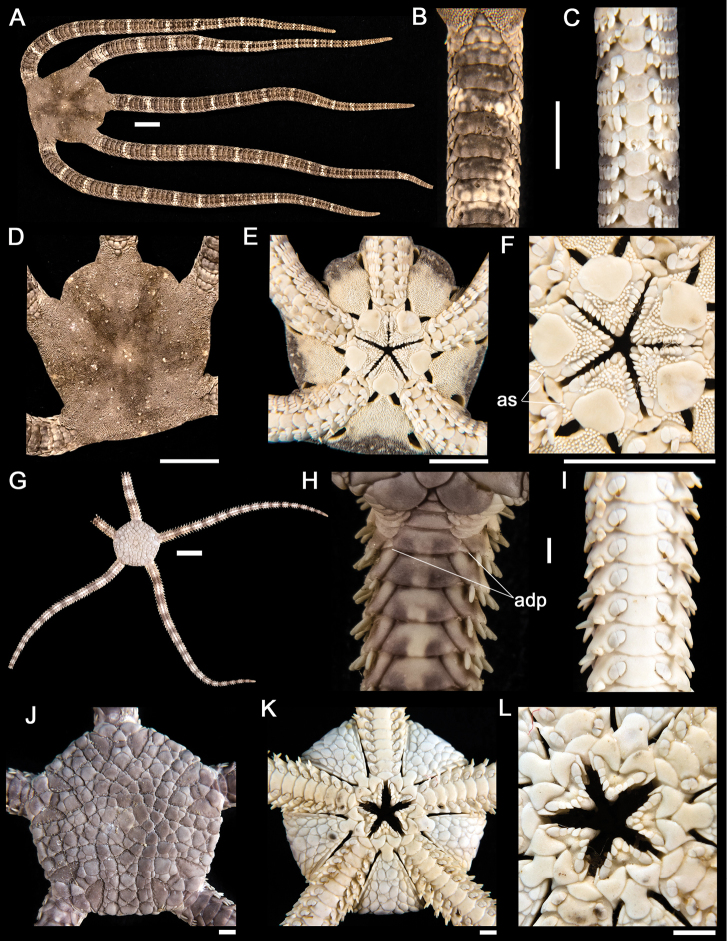
*Ophioderma* sp. 1. **A** dorsal view **B** dorsal view of the arm **C** ventral view of the arm **D** dorsal view of the disk **E** ventral view of the disk **F** jaw (ad = adoral shields). Scale bar = 5 mm. *Ophiolepis pacifica* **G** dorsal view. Scale bar = 5 mm **H** dorsal view of the arm (adp = accessory dorsal arm plates) **I** ventral view of the arm **J** dorsal view of the disk **K** ventral view of the disk **L** jaw. Scale bar = 1 mm.

**Description.** Disk pentagonal (dd = 8 to 16 mm). Radial shields covered by granulation (Fig. 7D). Dorsal and ventral side of the disk covered by fine, closely and rounded granulation (Fig. 7E). Oral shields large, heart-shaped and with three rounded lobes. Madreporite evident. Adoral shields not covered by granules, small and triangular. Eight to nine oral papillae on each side of the jaw, the outer one being the largest. Four teeth (Fig. 7F). Dorsal arm plates wider than long, rectangular with rounded edges (Fig. 7B). Ventral arm plates quadrangular and rounded. Ten to 11 short and blunt arm spines; all spines closely equal in size, except the lowest which is longer and thicker. Two tentacle scales, inner one larger and lanceolated. Four bursal slits per interradius (Fig. 7C). Dorsal side of the disk brown in color with some white marks (Fig. 7A, D); ventral side creamy but brown at margin (Fig. 7E). Dorsal arm plates brown with every five-seven plates with transversal and white bands. Ventral side creamy; the distal side of the ventral arm plates darker (Fig. 7E).

**Distribution.** In this study, *Ophioderma* sp. 1 was collected on coral reefs from Guerrero and Oaxaca, from 6.4 and 26 m depth.

**Remarks.** *Ophioderma* sp. 1 is a new species currently being described (pers. comm. FA Solís-Marín, UNAM, 2013). At first sight *Ophioderma* sp. 1 resembles *Ophioderma variegata* Lütken, 1856; however some significant different morphological characteristics were noted: 1) the oral shields of *Ophioderma* sp. 1 are heart-shaped, meanwhile in *Ophioderma variegata* are oval; 2) small adoral shields in *Ophioderma* sp. 1 but large in *Ophioderma variegata*; 3) *Ophioderma* sp. 1 has ten to 11 very short and blunt arm spines (occupying almost the half of the lateral arm plates) equal in size except the lowest arm spine that is longer and thicker, whilst *Ophioderma variegata* has seven-eight large arm spines equal in size and as long as the lateral arm plates; 4) all the collected individuals of *Ophioderma* sp. 1 displayed the color pattern mentioned in the species description section (brown in the dorsal side and creamy in the ventral side of the body), while *Ophioderma variegata* usually presents bright colors as pink, red, green and yellow (Verrill 1867, Koehler 1914, Caso 1951)*. Ophioderma* sp. 1 can be easily distinguished from *Ophioderma panamensis* and *Ophioderma teres* by its naked adoral shields. *Ophioderma* sp. 1 was collected under rocks.

**Collected material. GUERRERO: Zacatoso** (2 specimens, rock, 9.1 m, 01/06/2012, ICML-UNAM 10582); **El Chato** (1 specimen, rock, 13.7 m, 04/03/2009, ICML-UNAM 10189); **Palmitas** (1 specimen, rock, 6.4 m, 20/11/2011, ICML-UNAM 10459).

**OAXACA:** **Punto de Presión** (1 specimen, rock, 26 m, 22/04/2009, ICML-UNAM 10209); **El Faro** (1 specimen, rock, 23/04/2009, ICML-UNAM 10233; 1 specimen, rock, 13.5 m, 24/04/2012, ICML-UNAM 10519); **Copal** (1 specimen, rock, 9.1 m, 21/10/2011, ICML-UNAM 10444).

#### Family Ophiolepididae Ljungman, 1867

##### *Ophiolepis* Müller & Troschel, 1840

###### Ophiolepis pacifica

Lütken, 1856

http://species-id.net/wiki/Ophiolepis_pacifica

Figure 7G–L

####### Description.

Disk rounded (dd = 4 to 10 mm). Disk covered by large scales, surrounded by smaller scales of different sizes and shapes. There is a primary central scale surrounded by five oval scales. Radial shields triangular and distally separated by a row of scales; with a furrow that parallels the margin (Fig. 7J). The ventral interradial area is covered by scales smaller in comparison to the dorsal side (Fig. 7K). Oral shields pentagonal, longer than wide. Madreporite evident. Adoral shields large, triangular, not in contact. Four oral papillae at each side of the jaw; the outer one being the largest (Fig. 7K). Dorsal arm plates wider than long. Accessory dorsal arm plates restricted to the first arm segments (Fig. 7H).Ventral arm plates with distal margin rounded, wider than long. Four to five short and conical arm spines. Two tentacle scales closely equal in size (Fig. 7I). Dorsal side light brown in color; dorsal arm plates with darker transvers bands (Fig. 7G). Ventral side uniformly creamy-white in color (Fig. 7K).

####### Distribution.

Mexico, Costa Rica and Panama (Clark HL 1940, Verrill 1867, Honey-Escandón et al. 2008, Alvarado et al. 2010). In Mexico, from the Gulf of California (Sinaloa), Nayarit, Jalisco, Guerrero and Oaxaca (Caso 1951, Solís-Marín et al. 2005, Honey-Escandón et al. 2008, Granja-Fernández and López-Pérez 2011). From the intertidal zone to 30 m depth (Maluf 1988). In this study, *Ophiolepis pacifica* was collected on coral reefs from Guerrero and Oaxaca, from 4.9 to 26 m depth.

####### Remarks.

*Ophiolepis pacifica* was described by Lütken (1856) as *Ophiolepis* but Lyman (1865) transferred this species to the genus *Ophiozona* Lyman, 1865 based on the absence of the accessory or supplementary dorsal arm plates. Devaney (1974) studied specimens from the Gulf of California which revealed accessory dorsal arm plates; thus the genus *Ophiozona* was rejected and replaced again by *Ophiolepis*. We also found small accessory dorsal arm plates in the first arm segments of the collected material and were more evident in larger specimens. *Ophiolepis pacifica* was collected buried in sand and although Clark HL (1940) reported *Ophiolepis pacifica* in *Pocillopora* coral in Jasper Island (Costa Rica), we never found the species associated with coral. In the Eastern Pacific, it is common to find other species of this genus (*Ophiolepis crassa* Nielsen 1932, *Ophiolepis fulva* Clark HL, 1940; *Ophiolepis grissea* Clark HL, 1940; *Ophiolepis variegata* Lütken, 1856) associated with sandy and muddy bottoms (Verrill 1867, Ziesenhenne 1937, Clark HL 1940).

####### Collected material.

**GUERRERO:** **Morro del Cerro Colorado** (2 specimens, sand, 5.5 m, 31/05/2012, ICML-UNAM 10571); **Morros de Potosí** (4 specimens, sand, 06/03/2009, ICML-UNAM 10204).

**OAXACA:** **Puerto Angelito** (1 specimen, sand, 10 m, 23/04/2012, ICML-UNAM 10511); **Punto de Presión** (1 specimen, sand, 26 m, 22/04/2009, ICML-UNAM 10210); **El Faro** (1 specimen, sand, 22.9 m, 22/04/2009, ICML-UNAM 10219; 1 specimen, sand, 23/04/2009, ICML-UNAM 10237); **Estacahuite** (1 specimen, sand, 17/04/2008, MHN 005-4359; 1 specimen, sand, 19/02/2009, MHN 005-4322); **La Mina** (4 specimens, sand, 17/02/2008, MHN 005-4356); **Boquilla** (1 specimen, sand, 02/11/2007, MHN 005-4385); **San Agustín** (2 specimens, sand, 6.1 m, 15/09/2007, MHN 005-4333); **Riscalillo** (1 specimen, sand, 04/12/2008, MHN 005-4335); **Guerrilla** (2 specimens, sand, 4.9 m, 18/05/2012, ICML-UNAM 10541); **Copalita** (1 specimen, sand, 9.1 m, 18/05/2012, ICML-UNAM 10533).

## Discussion

This work represents the first effort for assessing the biodiversity of brittle stars associated with coral reefs in the Tropical Mexican Pacific. The effort covers approximately 1,200 km along the Pacific coast of Mexico. The 14 species of brittle stars collected in these ecosystems represents 22.2% of the total of species (63) previously reported from the Mexican Pacific (Honey-Escandón et al. 2008). The number of reported species in this study is higher in comparison to similar studies conducted in specific reef systems from the Mexican Pacific: Cabo Pulmo (eight species), Isla Isabel (five species), Bahía de Chamela (11 species), La Entrega (five species), and coral reefs from Guerrero and Oaxaca (12 species) (Cintra-Buenrostro et al. 1998, Benítez-Villalobos 2001, Zamorano and Leyte-Morales 2005, Ríos-Jara et al. 2008, 2013, Granja-Fernández and López-Pérez 2011). The numerical discrepancy of species among studies may be due to the larger number of reef systems visited (59) and the systematic sampling of independent substrata of the present study. Regarding the substrata, we collected on live and dead stony corals, gorgonians, rock, sand, algae, rhodoliths, and sponges, whereas previous studies focused only on coral, rock and/or sand (Cintra-Buenrostro et al. 1998, Benítez-Villalobos 2001, Zamorano and Leyte-Morales 2005, Ríos-Jara et al. 2008, 2013). In addition, this study was focused exclusively on collecting brittle stars rather than all echinoderms.

All the collected brittle stars have an Eastern Pacific biogeographic affinity except *Ophiactis savignyi* which is considered cosmopolitan (Hendler et al. 1995). The most conspicuous species were members of the genera *Ophiocoma* (*Ophiocoma aethiops* and *Ophiocoma alexandri*), *Ophiactis* (*Ophiactis savignyi* and *Ophiactis simplex*) and the brittle star *Ophiothrix (Ophiothrix) spiculata*. Ophiocomidae, Ophiotrichidae and Ophiactidae are the most specious families in tropical shallow waters as well as on coral reefs world-wide (i.e. Caribbean, Barbados, Mexico, Seychelles) (Taylor 1968, Clark AM 1976b, Lewis and Bray 1983, Hendler et al 1995, Benítez-Villalobos 2001, Ríos-Jara et al. 2013). On the contrary, the brittle stars *Ophiocnida hispida*, *Ophiophragmus papillatus* and *Ophioderma* sp. 1 were rare or least common in the study area.

Four out of the 14 collected species represent new distribution records for the Mexican Pacific: *Ophiocnida hispida* is a new record for Jalisco, *Ophiophragmus papillatus* for Guerrero, and *Ophiothrix (Ophiothrix) spiculata*, and *Ophiactis simplex* are new for Colima. As previously mentioned, the collection of *Ophiophragmus papillatus* specimens is notable given that the holotype is the only known specimen of the species (Ziesenhenne 1940). Clearly, *Ophiophragmus papillatus* is a rare species in the study area; as we only found three specimens during 70 field collecting trips. All *Ophiophragmus papillatus* collected were juvenile and we recommend carrying out further directed samplings trips in order to find the substrata where adults occur or inhabit. Regarding *Ophioderma* sp. 1, the species is morphologically similar to *Ophioderma variegata* but differences are enough to grant species status to the former.

Finally, our results suggest that species of the same genus tend to use and or inhabit the same substrata. For example, members of the genus *Ophiocoma* were only collected on live and dead stony coral, rocks, algae and rhodoliths. *Ophiactis* and *Ophiothrix* were recorded in live and dead stony coral, rocks, algae, rhodoliths and sponges, while *Ophioderma* were only found on rocks and in sand. Along the Mexican Pacific, all these substrata exist in areas other than coral reefs, making the identification key provided here an important potential tool for use in non-reef areas. It is also evident that corals are an important key habitat for juvenile and adult brittle stars of several genera and species in the Tropical Mexican Pacific. Studies suggest that coral reefs could provide refuge from predation, for reproduction and feeding for both brittle stars and other invertebrates (Hendler and Littman 1986, Spalding et al. 2001). Recently, corals and coral reefs in the Mexican Pacific have experienced a progressive increase in disturbance associated with anthropogenic activities (fisheries, aquaculture, agriculture, industrial development, tourism and recreation, harbor construction and maintenance, urban development, garbage presence, habitat destruction and wastewater discharges) (Reyes-Bonilla 2003, Ortiz-Lozano et al. 2005), and subsequent loss in coral cover (López-Pérez et al. 2012), with few signs of this deterioration slowing in the near future. The loss of coral reef-building species can reduce the three dimensional structure and functional integrity of the reef (Álvarez-Filip et al. 2013) affecting different brittle stars activities (e.g. reproduction, refuge), while eutrophication due to anthropogenic activities can increase the dominance and biomass of some species (e.g. suspension feeders) and reduce other (Duinevald et al. 1987). Consequently, the future for brittle stars in the Mexican Pacific may be threatened if coral substrata are further reduced or degraded.

## Supplementary Material

XML Treatment for Ophiocnida hispida

XML Treatment for Ophiophragmus papillatus

XML Treatment for Ophiothrix (Ophiothrix) rudis

XML Treatment for Ophiothrix (Ophiothrix) spiculata

XML Treatment for Ophiothela mirabilis

XML Treatment for Ophiactis savignyi

XML Treatment for Ophiactis simplex

XML Treatment for Ophionereis annulata

XML Treatment for Ophiocoma aethiops

XML Treatment for Ophiocoma alexandri

XML Treatment for Ophioderma panamensis

XML Treatment for Ophioderma teres

XML Treatment for Ophiolepis pacifica
